# Short‐Range Machine‐Learning Potentials for Aqueous Electrolyte Solutions

**DOI:** 10.1002/cphc.70385

**Published:** 2026-05-11

**Authors:** Lisa Hetzel, Christopher J. Stein

**Affiliations:** ^1^ Department of Chemistry and Catalysis Research Center TUM School of Natural Sciences Technical University Munich Garching Germany; ^2^ Atomistic Modeling Center Munich Data Science Institute Technical University of Munich Garching Germany

**Keywords:** alkali halides, electrolytes, long‐range interactions, machine‐learning potentials, molecular dynamics

## Abstract

Machine‐learning potentials (MLPs) extend the time and length scales of atomistic simulations, enabling the study of complex systems, such as electrolyte solutions. Yet most models face a tradeoff between accuracy, computational cost, and the ability to capture long‐range interactions. Large foundation models promise generality but often come with substantial overhead and energy demands. In contrast, compact, system‐specific models may offer a more sustainable path for large‐scale simulations. Here, we benchmark the MACE architecture on aqueous sodium chloride (NaCl) solutions, systematically varying model size and the level of equivariance to assess their effect on accuracy, stability, and efficiency. We find that predictive accuracy of the investigated MLPs has little influence on key physical observables considered here but is crucial for stability, highlighting the potential of minimal, dedicated models for efficient simulations of electrolyte solutions.

## Graphical abstract

Machine‐learning potentials enable simulations beyond the reach of *ab initio* methods. We benchmark small MACE models on aqueous sodium chloride (NaCl), varying size and equivariance to probe accuracy, stability, and efficiency. Model accuracy has little impact on observables but is essential for stability, highlighting the promise of minimal, dedicated models for large‐scale simulations.

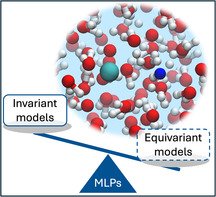

## 1 Introduction

Since the advent of machine‐learning potentials (MLPs) with the introduction of high‐dimensional neural network potentials (HDNNPs) by Behler and Parrinello in 2007 [1], numerous different architectures, including Gaussian approximation potentials [2], neural networks [1, 3], and message‐passing neural networks (MPNNs) [4, 5, 6] have been introduced. This led to a breakthrough in the time and length scales accessible to simulations, and many complex systems, such as interfaces and/or electrolyte‐containing solutions (see, e.g., Refs. [7, 8, 9, 10, 11, 12, 13, 14]), can now be studied with high accuracy.

A central approximation underlying most MLPs is the locality of atomic environments: each atom is described by a cutoff‐based descriptor, and its energy is predicted from its local environment. While this maintains scalability, it limits the description of long‐range interactions or charge‐transfer processes. To address this, several models have been developed that explicitly incorporate long‐range electrostatics, for example, via charge equilibration [15], Wannier‐based descriptors [16], spatio‐spectral graph neural networks [17], or latent Ewald summation schemes [18]. These extensions improve accuracy in cases where long‐range interactions are essential, such as vapor–liquid interfaces or the vapor phase of water [19, 20], but inevitably increase computational cost.

MPNNs, such as MACE [21] or NequIP [4], offer a natural compromise. By passing messages across several layers, they implicitly extend the effective interaction range while retaining computational efficiency [5, 22]. With typical cutoff radii of around 6 Å and one to three message‐passing layers [23], these models can already capture a large fraction of the relevant interactions in liquids, where intermolecular distances are on the order of 3 Å [24]. In particular, in bulk aqueous electrolyte solutions, long‐range electrostatics are strongly screened by solvent molecules [25], making short‐range MPNNs attractive candidates for modeling such systems.

Beyond accuracy, however, cost remains a central challenge for MLPs. On the one hand, evaluating energies and forces becomes more expensive as model complexity increases. On the other hand, generating reference training data often represents a major bottleneck, since it requires a large number of electronic‐structure calculations. To alleviate the training‐data bottleneck in specific application domains, large foundation models have recently been developed. These models aim to provide a general‐purpose force field covering broad chemical and structural space, often across most of the periodic table. Recent examples are the UMA [26] model by Meta, CHGNet [27], or the MACE‐MP‐0 [28] foundation models, among others [29, 30, 31, 32, 33, 34]. These general‐purpose models can then be fine‐tuned for a dedicated system [35]. However, since these models usually span a very diverse dataset, they need an enormous number of free parameters. Hence, they also increase computational cost and memory demand, which can hinder the main motivation for using an MLP, i.e., being able to run large‐scale simulations fast. Especially when graphics processing unit (GPU) resources are limited, this can limit the accessible system sizes and simulation timescales. While efforts are also being made to distill smaller, dedicated models out of large foundation models [36, 37], we here pursue the complementary strategy of training explicit, system‐specific MACE models. We investigate how well small, dedicated models can describe aqueous electrolytes by analyzing numerical error measures and examining how fit accuracy translates into the reliable prediction of computed static and dynamic properties. Hence, this study will provide guidelines on how to select models efficiently for future applications.

We selected MACE [21] as a recent MLP, including equivariant higher‐order message passing, which shows excellent data efficiency and accuracy [23]. MACE uses the atomic cluster expansion (ACE) [38] for message construction. In ACE, a many‐body expansion of the energy is employed. After applying a so‐called density trick, the tensor product can be exploited such that the evaluation scales linearly with the body order and system size. Therefore, descriptors of higher body order become feasible, contrasting traditional MLP descriptors, such as smooth overlap of atomic positions (SOAP) [39] or atom‐centered symmetry functions [1], where only body orders up to three are viable. This results in higher accuracy and more data‐efficient fitting, enabling linear regression in the original ACE formulation [38]. MACE employs the aforementioned approach for the construction of higher‐order messages. These are embedded into an equivariant message‐passing scheme similar to NequIP [4]. The introduction of equivariance has been shown to improve the accuracy and data‐efficiency of the fit [4, 5]. Moreover, one important hyperparameter is the number of uncoupled feature channels (k) for the message passing. In MACE, these channels correspond to independent ACE basis functions prior to body‐order coupling and, therefore, directly determine the size of the latent feature space and the model's representational capacity.

In this study, we systematically investigate the balance between accuracy and computational efficiency for compact, system‐specific MACE models. By varying the number of uncoupled channels, which defines the model size, and the equivariance level ($L_{\text{max}}$, 0 for an invariant and 1 for an equivariant model), we explore how architectural choices affect predictive accuracy, extrapolation behavior, and stability. Using sodium chloride (NaCl) solutions as a well‐characterized benchmark system, we assess whether small MACE models without explicit long‐range corrections can reliably capture static and dynamic physical properties at comparably low computational cost for such isotropic systems.

## 2 Results and Discussion

### 2.1 Construction of the MLPs

The first step in constructing an MLP is to generate high‐quality reference data that adequately covers the relevant configuration space. Benchmarking the underlying electronic‐structure method is therefore essential. However, this creates a contradiction, as many physical properties of interest are not directly accessible via *ab initio* methods due to the required long sampling time. Hence, MLPs can also serve as tools to benchmark electronic‐structure methods themselves (see, e.g., Ref. [13]). The main challenge for the reference method for aqueous NaCl is to accurately capture both ionic and aqueous interactions, which have been addressed in several studies [13, 40, 41, 42]. Such benchmarking is not the focus of our study. We hence chose the revPBE0‐D3 functional, which has been demonstrated to reliably reproduce experimental properties of bulk aqueous systems [43, 44]. Our resulting dataset contained roughly 40,000 structures, corresponding to 20 ps of sampling. We chose a plane‐wave cutoff of 800 Ry for sampling to make the simulation feasible. However, we stress that this is far from converged. In fact, we did not even reach converged forces on the sodium forces at several thousand Ry, which is also computationally infeasible (see Supporting Information (SI)). Hence, we switched to the Gaussian and augmented plane‐wave (GAPW) [45, 46] method in CP2K [47]. Consequently, a subset of the initial trajectory was recomputed using a converged plane‐wave cutoff to ensure accurate ionic forces (see Methods). Converging the plane‐wave cutoff was critical for achieving stable MLP‐based simulations.

### 2.2 Numerical Validation

**FIGURE 1:**
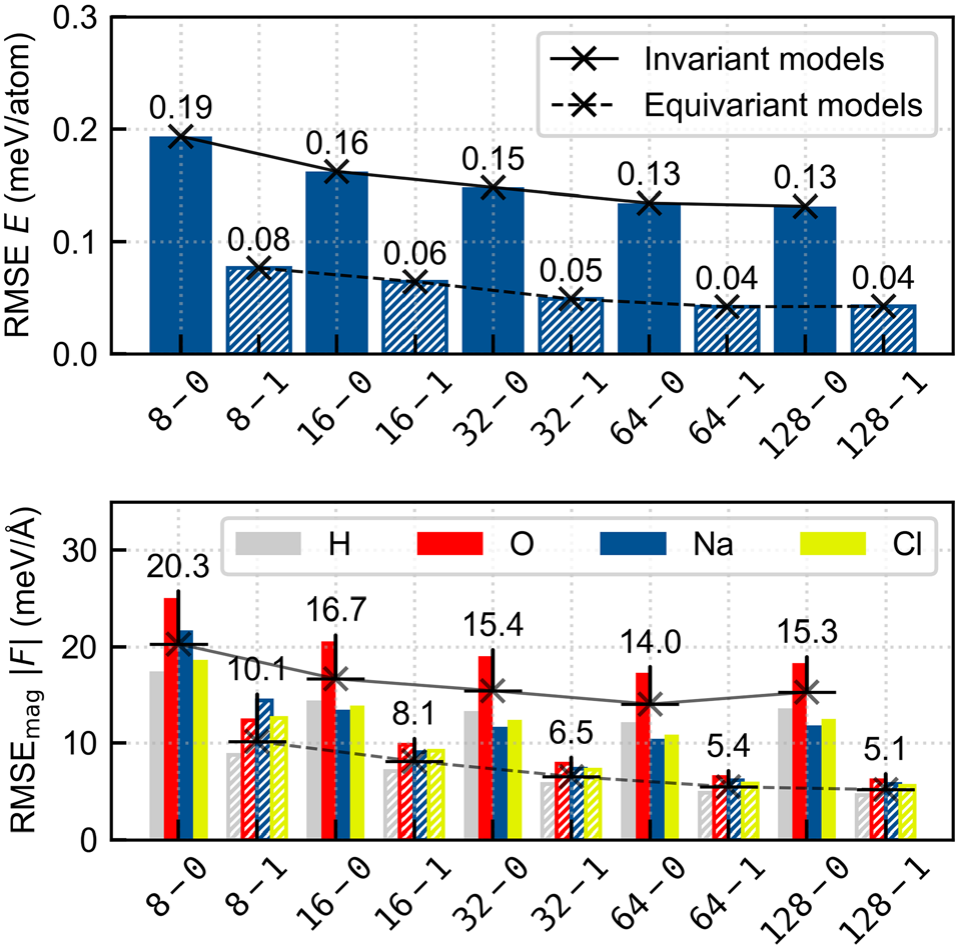
Numerical validation of trained MLPs with varying combinations of k and $L_{\text{max}}$, where we employ the same nomenclature as in Ref. [23]: $k-L_{\text{max}}$. The upper panel shows RMSEs for the energies E, and the lower panel shows the element‐wise and averaged RMSEmag of the forces F for the investigated aqueous NaCl system.

We trained several MACE potentials using the aforementioned datasets and performed numerical validation. We systematically varied the level of equivariance ($L_{\text{max}}$) and the number of uncoupled channels (k), as both hyperparameters significantly affect the computational cost of training and simulation [23]. We computed root‐mean‐squared errors (RMSEs) using the RMSEmag for force vectors following Ref. [48]. Figure 1 shows the resulting error metrics for different models. We include the commonly employed component‐wise force RMSEs in the SI for better comparability. In the following, we use the same nomenclature as in Ref. [23]: k‐$L_{\text{max}}$, e.g., 32–0 for an invariant model with 32 uncoupled channels.

In general, increasing complexity leads to consistent improvements in both energy and force predictions. Introducing equivariance reduces errors by approximately 30%–50%, in line with the findings by Batzner et al. [4]. In contrast, increasing k alone yields more modest gains. Energy errors plateau and force errors even increase beyond k=64, suggesting that the model's capacity is exceeded and that further increasing its complexity leads to overfitting. We also note that the most accurate invariant 64–0 model still does not outcompete the smallest equivariant 8–1. Furthermore, the reported errors are in good agreement with what is the current state‐of‐the‐art; see, e.g., Ref. [23]. As already outlined in the introduction, the motivation behind choosing the smallest model possible is to reduce cost and, hence, to be able to run large‐scale simulations. The accompanying computational cost for both training and evaluation of the MLP is discussed in the final paragraph of this section.

### 2.3 Stability of MLPs and Extrapolation Analysis

**FIGURE 2:**
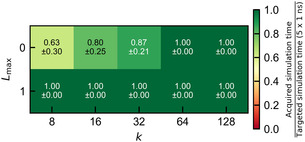
Simulation stability across models of varying architectural complexity for aqueous NaCl. The heatmaps show the average fraction, with its standard deviation, of five different 1 ns trajectories successfully simulated for each model, with models arranged by the number of uncoupled channels (k) and the maximum level of equivariance ($L_{\max}$). Color intensity indicates stability, with green representing complete runs. Numerical values denote the mean ± standard deviation across five runs. The simulations are terminated when an atom moves more than one box length during one step, which would lead to unphysical structures.

We used the obtained MLPs to run simulations and analyze the stability of the trajectories. Therefore, for each MLP, we ran five trajectories for 1 ns each after an initial equilibration of 100 ps. Figure 2 shows the fraction of successful simulation time, averaged over five simulation runs starting from different initial configurations. As a criterion for instability, we choose the unphysical situation in which an atom is moved by more than one box length in a single integration step.

We observe that all equivariant MLPs produce stable simulations for this simulation length. In contrast, the invariant models with k≤32 show substantial instabilities, although the smallest 8–0 model still reaches more than 3 ns on average. This increased tendency to provide unstable trajectories, with a smaller number of uncoupled channels, is likely a direct result of inaccurate force predictions, which first result in configurations with unphysically short interatomic distances, ultimately leading to extremely large forces resulting from the steep repulsive potential. Alternatively, these instabilities may reflect differences in the extrapolation behavior of the models or limitations in the training data. To better understand their origin, we investigated whether the observed failures in the invariant models arise from insufficient coverage of the relevant configuration space during training, or from extrapolation‐induced errors in force predictions that initiate a cascade of unphysical configurations. Understanding this distinction is important, since iterative retraining to fix instabilities can be prohibitively expensive, while simply using larger models is also costly. Knowing whether the failures stem from extrapolation or other effects is thus crucial for choosing an efficient path toward more robust models.

We used the kernel of SOAP vectors K as a similarity measure for different configurations as defined in Ref. [49],

(1) $$K={\left({\left|k\left(p_{0}\cdot p_{i}\right)\right|}^{\zeta },{\left|k\left(p_{0}\cdot p_{j}\right)\right|}^{\zeta },\ldots \right)}^{T}$$

where $p_{0}$ is the SOAP vector of a new configuration, $p_{i}$ the ith configuration in the training dataset, and ζ a positive integer for which we used the same value as in Ref. [49] (see Computational Methods for details). We computed an extrapolation score defined as

**FIGURE 3:**
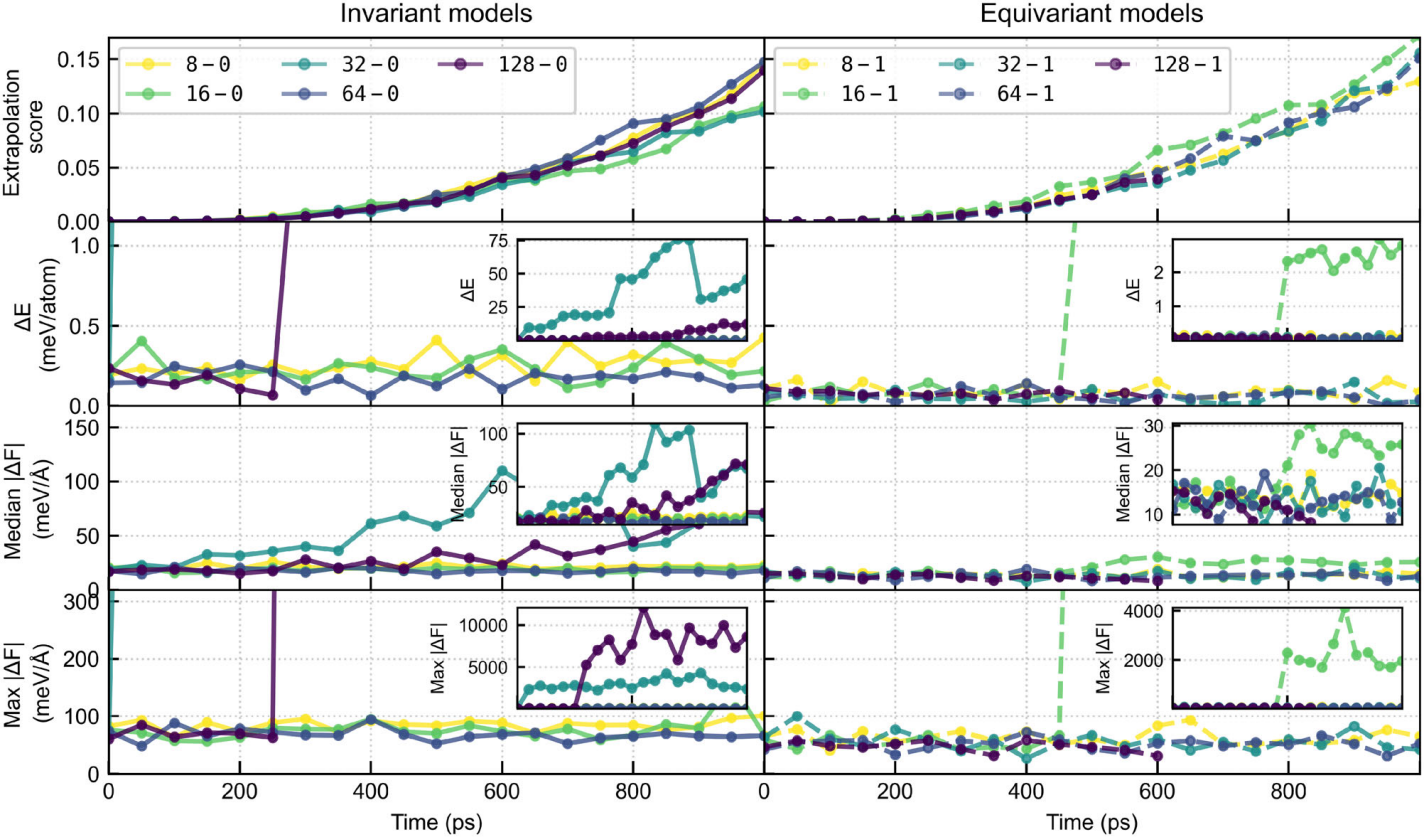
Extrapolation analysis of a representative trajectory for each model, all initiated from the same starting configuration. Each model was equilibrated independently, leading to decorrelated trajectories. Shown are the extrapolation score (Equation (2), top panel), the energy difference ΔE (second panel), the median magnitude of the atomic force error (third panel), and the maximum magnitude of the atomic force error (bottom panel) with respect to DFT for all model architectures. Results for invariant models ($L_{\text{max}}=0$) are displayed in the left column, and for equivariant models ($L_{\text{max}}=1$) in the right column.

(2) Extrapolation score=1−max(K)

such that the training configuration with the highest similarity determines the score. Additionally, we computed the energies and forces with the density functional theory (DFT) reference method for one 1 ns trajectory and the resulting error of the MACE model. The results are shown in Figure 3, where we also report errors compared to reference points computed at the same level as the training data.

We observe that for all models, the extrapolation score increases over time, consistent with what is seen in other trajectories (see SI). In contrast, the prediction accuracy differs across model architectures. The invariant 32–0 and 128–0 models exhibit energy deviations considerably exceeding their test set errors (0.15 and 0.13 meV/atom; see Figure 1), reaching values above 10 meV/atom. Although the median force error remains initially stable, the maximum atomic force errors grow sharply, indicating that local force predictions can become unphysical. These erroneous forces propagate through the local environment and eventually drive an increase in the median force error. Importantly, the extrapolation score alone does not capture this mode of failure.

The fact that the energy error remains essentially constant for some models suggests that the training dataset is sufficiently converged and that the observed drifts mainly reflect architectural differences. In particular, the invariant models appear more prone to local force errors, which explains their instabilities. By contrast, the equivariant models not only show generally lower errors in line with their test set performance, but also display greater stability; aside from a drift in energies for the small 16–1 model, the energy error stays below 3 meV/atom and does not increase further. This indicates that equivariant models may recover better from perturbations in force prediction.

In the following section, we will assess how these issues manifest in physical observables, as this ultimately determines model reliability.

### 2.4 Physical Validation

**FIGURE 4:**
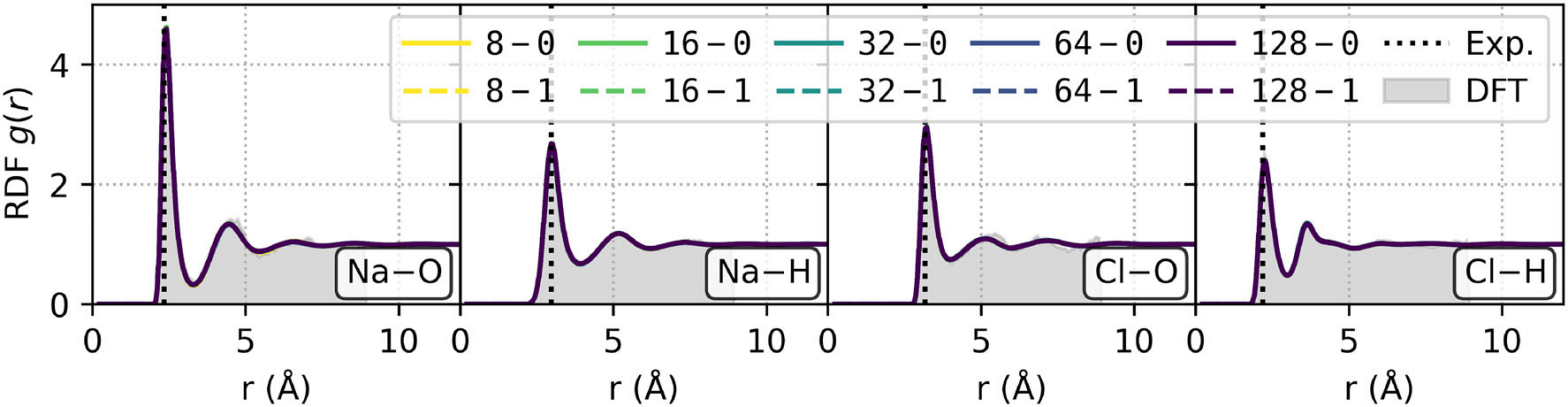
Pairwise RDFs g(r) of sodium and chloride solvation shells are shown for simulation trajectories obtained with MLPs of different architectures. For comparison, RDFs from the underlying DFT training dataset (gray) and the experimental reference [50] for the position of the first peak (dashed vertical line) are also included.

To assess how fitting accuracy translates into physically meaningful properties, we first analyze static structural observables—specifically, pairwise radial distribution functions (RDFs). Figure 4 presents the RDFs characterizing the solvation shells around each ion. For reference, we also include DFT data from the full trajectory. While this was computed using a non‐converged plane‐wave cutoff, previous work by O’Neill et al*.* [13] has shown that structural observables, such as RDFs, remain unaffected. All MLPs, in contrast, were trained on data computed with a fully converged cutoff to obtain stable potentials.

We observe that the RDFs computed from simulations, using different model architectures, are in excellent agreement with one another despite differing fit accuracy. Moreover, they closely match the DFT reference data from the original dataset. This shows that the fit error does not significantly influence the accuracy of static properties, such as RDFs. In addition, the RDF peak positions closely match experimental data [50], supporting the reliability of the underlying DFT functional in capturing the essential solvation structure.

**FIGURE 5:**
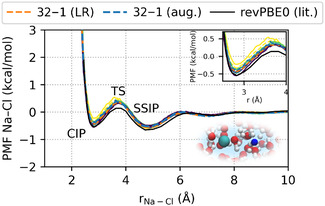
Potential of mean force (PMF) obtained from inversion of the pairwise RDF, $\mathrm{PMF}=-RT\ln\;g_{\mathrm{Na}\text{‐}\mathrm{Cl}}(r)$, for all model architectures. Additionally, we include results from a 32–1 model trained on an augmented dataset with larger simulation boxes, with and without long‐range electrostatic corrections (see Methods). The color and line‐style coding for the short‐range models follows the legend in Figure 4. Literature reference curves are taken from Ref. [13].

In addition, we show the potential of mean force (PMF) in Figure 5 describing the cation–anion interaction, which is based on the pairwise cation–anion RDF (see SI for information on the calculation of the PMFs) and provides insight into the long‐range behavior of the system. The PMFs obtained from different model architectures also agree closely, with deviations typically below 0.2 kcal/mol. A notable exception is the 8–0 model, which predicts a shallower contact ion pair (CIP) minimum and a higher barrier for the transition to the solvent‐separated ion pair (SSIP). However, only 3.15 ns of the targeted 5 ns trajectory could be generated for this model due to the instability discussed above, so the observed deviations may partly reflect insufficient sampling rather than genuine model inaccuracy. Moreover, we obtain qualitative agreement with the PMF reported by O’Neill et al*.*, computed using the same DFT reference method. The remaining differences may arise from the use of different salt concentrations. The PMFs also highlight the long‐range nature of ion–ion interactions: the NaCl potential converges to zero at around 8–10 Å, indicating effective screening in aqueous solution. To explicitly assess the role of long‐range electrostatics, we additionally performed simulations using a 32–1 model trained on an augmented dataset containing larger simulation boxes, with and without an explicit long‐range correction using the latent Ewald summation [18]. The resulting PMFs are virtually identical to those obtained with the corresponding short‐range model, both in the depth of the contact ion pair minimum and in the asymptotic decay at larger separations. Consistent behavior is also observed in the ion–ion RDFs (Na–Na and Cl–Cl), which are reported in the Supporting Information (see Figure S30) and show identical long‐range decay for short‐range and long‐range simulations. These findings are consistent with classical molecular dynamics (MD) and *ab initio* molecular dynamics (AIMD) studies [13, 51, 52] and support the applicability of short‐range MPNNs for modeling dilute electrolyte solutions.

**FIGURE 6:**
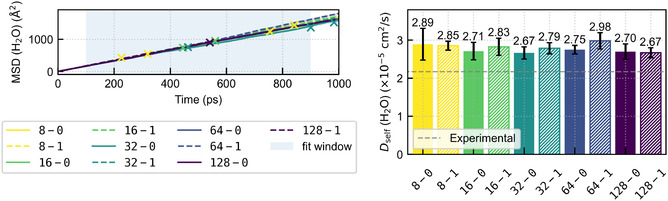
Mean‐squared displacement (MSD) of all water molecules (left panel) and corresponding self‐diffusion coefficients (right panel) obtained from molecular dynamics simulations with different model architectures. The MSD curves represent the mean over five independent trajectories for each model, where shaded bands indicate the statistical uncertainty of the mean, and crosses mark trajectories that terminated before the end of the simulation time. Self‐diffusion coefficients were obtained from linear fits to the MSD in the diffusive regime (100≤t≤900 ps) using a block‐averaging procedure with 100 ps blocks. The reported values and uncertainties correspond to the mean and standard error over all block estimates pooled across trajectories (see SI for details). The gray dashed line indicates the experimental reference value at 1.0 mol/kg from Ref. [53].

Moreover, self‐diffusion coefficients of water molecules were computed via the Einstein relation (see SI), employing the mean‐squared displacement (MSD) for all model architectures. While ionic diffusivities or conductivities are more directly related to transport properties, their reliable determination would require substantially longer simulation times than considered here. We therefore use the water self‐diffusion coefficient as a proxy to enable a consistent model‐by‐model comparison of a dynamical observable. Figure 6 shows the MSD for all trajectories and the resulting self‐diffusion coefficients averaged over all trajectories.

Within the estimated uncertainties, the coefficients obtained from all models are in very good agreement. We observe a slight tendency for the equivariant models to yield higher diffusion coefficients, possibly due to a smoother potential energy surface (PES). The general agreement among all the models is somewhat surprising, as dynamic quantities are expected to be more sensitive to fitting errors, as also shown in recent comparative studies of different MLPs [54, 55]. Hence, we conclude that, within the range of the observed fitting errors, dynamic quantities remain unaffected owing to the overall high accuracy of the MACE architecture, even though instabilities were observed for the invariant models.

Nevertheless, our results systematically overestimate the experimental self‐diffusion coefficients. It is well established that the presence of NaCl decreases the diffusion of water molecules, whereas, for example, potassium ions increase it [56]. This discrepancy can be attributed to the limitations of the underlying DFT reference method. Indeed, several studies demonstrate that wave‐function‐based approaches are required to achieve quantitative agreement with experiment [13, 57]. We therefore refer to the extensive literature on the accurate simulation of aqueous electrolyte solutions; see, e.g., Refs. [13, 42].

### 2.5 Computational Cost

**FIGURE 7:**
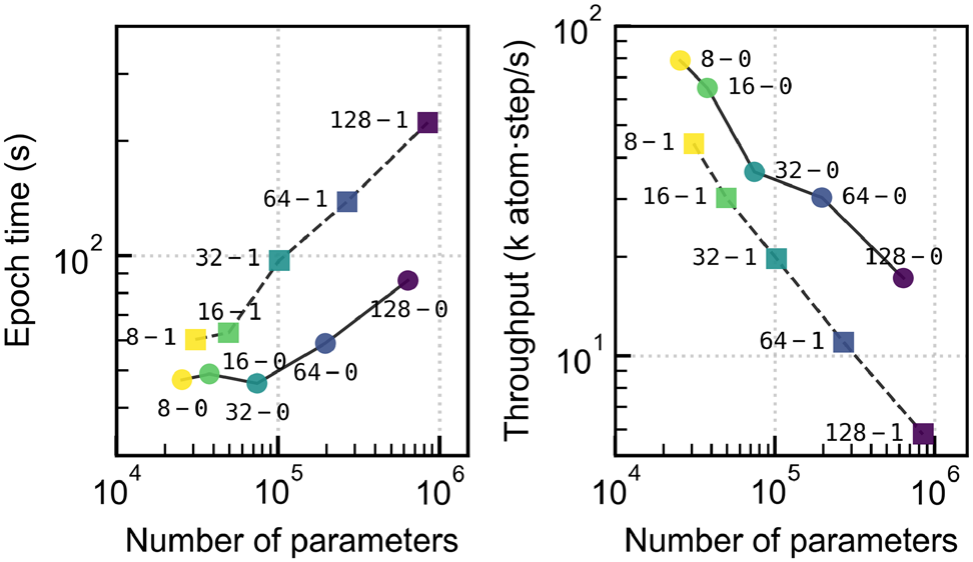
Computational cost of model training and MD simulations correlated with the number of free parameters of the respective model architectures. The left panel shows the time required for a single training epoch, which depends on the size of the training dataset and is averaged over all epochs. The right panel reports the number of atoms and simulation steps that can be evaluated per second, averaged over all conducted simulation runs. For details on the used hardware; see Methods.

Finally, we discuss the computational cost associated with both training the model and running the simulations. In Figure 7, we show the cost associated with training the model and evaluating the PES correlated with the model size, namely, the number of free parameters.

Training cost is particularly relevant in the context of active learning. As expected, equivariant models are generally more expensive due to the increased number of parameters and higher‐order tensor products. For invariant models, cost increases noticeably from the 32–0 model onward, while for equivariant models, the rise begins with the 16–1. For the smallest models, overhead likely dominates the cost. More critical, however, is the evaluation cost during MD, where throughput consistently decreases with model size, and equivariant models remain more demanding. Notably, the 16–1 model has a cost comparable to the 64–0 invariant model while achieving superior fit accuracy. Thus, even though physical observables do not differ significantly between models, small equivariant architectures, with low k, offer a favorable tradeoff between cost, stability, and accuracy—especially when training data are limited or expensive and active learning (see, e.g., Ref. [58]) is infeasible. In addition to wall time, memory requirements can also become limiting, reinforcing the need to favor smaller model architectures.

## 3 Conclusion

In this study, we systematically investigated the impact of model size and equivariance in the MACE architecture for aqueous NaCl. Our results indicate that equivariance plays a more decisive role than the number of parameters, with even relatively small equivariant models achieving higher accuracy and stability than much larger invariant counterparts. By contrast, the increase in model size through additional parameters alone was found to have a comparatively minor effect.

We further observe that many physical observables are only weakly sensitive to predictive accuracy, suggesting that invariant or otherwise compact models may still be suitable in applications where short simulations or reduced computational cost are prioritized. Encouragingly, instabilities tend to appear as simulation breakdowns before they affect the evaluation of physical quantities, which implies that model scaling can be pursued with reduced risk of drawing misleading conclusions from apparently stable trajectories in the case of aqueous electrolytes. Instabilities may eventually be ruled out by employing sophisticated pretraining strategies using computationally efficient methods, such as fixed‐charge force fields [59].

Finally, our analysis of the potential of mean force suggests efficient screening in NaCl solutions. An explicit comparison between short‐range and long‐range‐augmented models shows no qualitative or quantitative differences in the resulting PMFs or ion–ion RDFs. While the effect of including long‐range electrostatics on ionic transport properties, such as ionic conductivities, was not addressed in the present work, this represents an interesting direction for future investigations.

## 4 Computational Methods

### 4.1 Reference Data

The reference dataset was generated through AIMD simulations at DFT level using the program package CP2K [47] Version 8.2. Full simulation details are provided in the SI. The energies and forces for boxes containing one or two NaCl ion pairs and 64 water molecules were recomputed for a subset of the initial dataset with a converged plane‐wave cutoff. We used the revPBE0 [60] hybrid functional with D3 dispersion correction [61], the MOLOPT TZV2P [62] basis set, and Goedecker–Teter–Hutter (GTH) pseudopotentials [63, 64, 65]. We employed a plane‐wave cutoff of 2000 Ry with the GAPW method [45, 46] as implemented in CP2K to converge the plane‐wave cutoff. A detailed discussion of the plane‐wave cutoff convergence and more information on the dataset generation can be found in the SI.

In addition to this reference dataset, an augmented set of configurations was generated to assess the role of long‐range interactions. For this purpose, larger simulation boxes containing one and two NaCl ion pairs in 146 or 149 water molecules, respectively, were sampled from classical MD simulations. The resulting configurations were subsequently recomputed at the same DFT level as described above to obtain energies and forces. Further details of the structure‐generation protocol are provided in the SI.

### 4.2 Construction of the MLPs

We constructed the training dataset from 1500 structures containing a single NaCl ion pair and 750 structures containing two ion pairs, in addition to 64 water molecules. The side length of the cubic simulation box is 12.484 Å for the lower concentration and 12.553 Å for the higher concentration of the NaCl solution. In addition, 32 single‐water structures, generated by scanning along the normal modes, were included to improve the stability of the MLP. For validation, 5% of the training data was used. The test set comprised 750 single‐ion‐pair structures and 375 two‐ion‐pair structures, none of which were used during training. We trained 10 MACE [21] version 0.3.5 models in total, with k=8,16,32,64,128 and $L_{\text{max}}=0,1$. The cutoff radius was set to $r_{\mathrm{cut}}=6$Å, with two layers spanning a total range of 12Å during message passing. All other hyperparameters were kept fixed across model architectures and are provided in the SI.

To assess the impact of long‐range interactions, we trained two additional models using an augmented dataset derived from classical molecular dynamics simulations. This augmented set includes 90 structures with one ion pair and a box side length of 16.742 Å, and 84 structures with two ion pairs and a box side length of 16.674 Å. The test dataset was kept identical to that used for the short‐range models. Using MACE version 0.3.14, we trained two models with k=32 and $L_{\text{max}}=1$: one short‐range model and one model augmented with an explicit long‐range electrostatic correction using latent Ewald summation [18]. The training procedure and all remaining hyperparameters were kept identical to those used for the short‐range models.

### 4.3 MLP Simulations

The simulations with the trained MLPs were primarily run using the MD engine LAMMPS [66] integrating MACE. A time step of 0.5 fs was used with a Nosé–Hoover thermostat to run simulations within the canonical (NVT) ensemble at 300 K. The time constant of the thermostat was set to 50 fs. We employed a box with 512 water molecules and eight ion pairs and a box size of 24.968 Å under periodic boundary conditions. Five initial configurations were randomly sampled from the test dataset and equilibrated for another 100 ps. All production runs were conducted for 1 ns.

For the long‐range interaction study, the MD simulations were performed using the Atomic Simulation Environment (ASE) [67, 68]. These simulations used the same starting configurations, time step, and target temperature but employed a Langevin thermostat with a friction coefficient of $5\times 10^{-3}$ fs−1.

### 4.4 Extrapolation Analysis

For the extrapolation analysis, we used an adapted version of the mlptrain [49] package. Each configuration was described via a periodic atomwise SOAP descriptor with a cutoff of 6 Å, the maximum degree of the radial basis functions $l_{\text{max}}=8$, and the number of radial basis functions $n_{\text{max}}=8$. Following Ref. [49], the kernel was computed with an exponent of ζ=4.

### 4.5 Hardware Specifics

We trained our models on NVIDIA A100 GPUs, each equipped with 80 GB of memory and delivering up to 19.5 TFLOPS of single‐precision performance.

## Funding

This study was supported by Deutsche Forschungsgemeinschaft (Grant 2089/1‐390776260 and 443703006).

## Conflicts of Interest

The authors declare no conflicts of interest.

## Supporting information

Supplementary Material

## Data Availability

The training data, test data, CP2K input and trajectory snapshots are available as a Zenodo repository https://doi.org/10.5281/zenodo.18108882.
