# Supplementary material for: Short‐Range Machine‐Learning Potentials for Aqueous Electrolyte Solutions
Source: Chemphyschem. 2026 May 11;27(9):e70385. doi: 10.1002/cphc.70385 (PMC13160254; doi:10.1002/cphc.70385)
Supplement: Supplementary file 1 — Supplementary Material [file CPHC-27-e70385-s001.pdf]

Supporting Information for: Short-range machine-learning potentials for electrolyte solutions

Lisa Hetzel[†], Christopher J. Stein[†,*]

[†]TUM School of Natural Sciences and Catalysis Research Center,
Department of Chemistry
Technical University of Munich
Lichtenbergstr. 4, 85748 Garching, Germany
Emails: lisa.hetzel@tum.de; christopher.stein@tum.de

1 Further computational details of the Reference data set

The reference data set was generated in two steps. In the first step, we ran *ab initio* molecular dynamics (AIMD) simulations, and in the second step, we recomputed a data set with a converged plane wave cutoff. The details are explained in the following sections.

1.1 Simulation set up for the reference data generation based on AIMD

The simulation boxes comprised 64 water molecules and one or two sodium chloride ion pairs. The box sizes are listed in Table S1. Initial configurations were generated using the program package `packmol`[S1]. The initial configurations were equilibrated for 1 ns with a time step of 2 fs at 300K in the NVT ensemble using the classical molecular dynamics (MD) program package GROMACS[S2] in conjunction with the non-polarizable OPSLAA[S3] force field. Subsequently, a production run was performed under the same conditions for 1 ns. From this run, four initial configurations with different distances between the ions were chosen as starting configurations for the AIMD simulations. These configurations included the formation of the contact ion pair (CIP) and solvent-separated ion pair (SSIP), ensuring a more comprehensive exploration of the configuration space.

Table S1: Set up of the simulation boxes of the AIMD simulations of the aqueous NaCl solution under periodic boundary conditions.

Number of ion pairs	Box side length [Å]
1 NaCl	12.484
2 NaCl	12.553

1.2 AIMD simulations for structure generation

The AIMD simulations were performed in the NVT ensemble at 300K with a time step of 0.5 fs and the CSVR[S4] thermostat. All systems were equilibrated for at least 3 ps, with the coupling constant of the thermostat being set to 30 fs. The trajectories were started from different configurations sampled from classical molecular dynamics simulations (see section 1.1). For the production run, a coupling constant of 1 ps was used. In total, trajectories of at least 20 ps were obtained such that at least 40.000 structures per system were generated for the simulation boxes containing one ion pair. For the simulation boxes containing two ion pairs, at least 5000 structures were generated.

The AIMD simulations were carried out with the same electronic-structure setup as used for the final data set, but with a reduced plane wave cutoff of 800 Ry to keep the dynamics computationally feasible. A subset of the resulting snapshots was later recomputed with a converged cutoff, as described in Section 1.3.

1.3 Computation of energies and forces

The data set was based on the AIMD trajectories described above, which were generated at the revPBE0-D3[S5–S7] level of theory using the MOLOPT TZV2P[S8] basis set and GTH pseudopotentials[S9–S11]. For sodium, the $2s$ and $2p$ core electrons were treated explicitly in addition to the $3s$ electron to avoid the issue of non-linear core and valence electron exchange/correlation.[S12] Here we used the Gaussian plane wave (GPW)[S13] method with a plane wave cutoff of 800 Ry during the dynamics. To remove cutoff-related artifacts, the energies and forces were recomputed for a subset of configurations with the identical electronic-structure setup but employing the GAPW scheme[S14,S15] and a converged cutoff of 2000 Ry (see discussion below). Moreover, a relative cutoff of 80 Ry and 5 multigrids were employed. To avoid self-interaction between periodic images and to reduce the computational cost of the hybrid functional calculations, a truncated Coulomb operator with a cutoff radius of 6 Å was applied to the Hartree–Fock exchange term, as implemented in CP2K. We note, that the Hartree (electrostatic) contribution was evaluated using the full long-range Coulomb interaction under periodic boundary conditions. Furthermore, the auxiliary density matrix

method[S16] was utilized to speed up calculations of the Hartree-Fock exchange. Therefore, the cpFIT3[S16] auxiliary basis set was employed.

1.4 Discussion of the plane wave cutoff

As already discussed by O'Neill *et al.*[S17], the forces on the sodium atoms require a large plane wave cutoff using the Gaussian plane wave method[S13] (GPW) commonly used by CP2K[S18] in conjunction with DFT calculations. Therefore, we performed convergence tests on all systems. Figure S1 shows the forces on all four atom types for the aqueous NaCl on a random snapshot. The forces on the sodium cation shown in Figure S1 do not converge for a cutoff smaller than 5000 Ry. This can be attributed to the narrow Gaussians of the basis set, requiring a fine grid. This issue can be resolved using the GAPW method[S14,S19]. Though it comes with more approximations, using a plane wave cutoff of up to several thousand Rydberg becomes unfeasible, such that the GAPW provides a reasonable compromise.

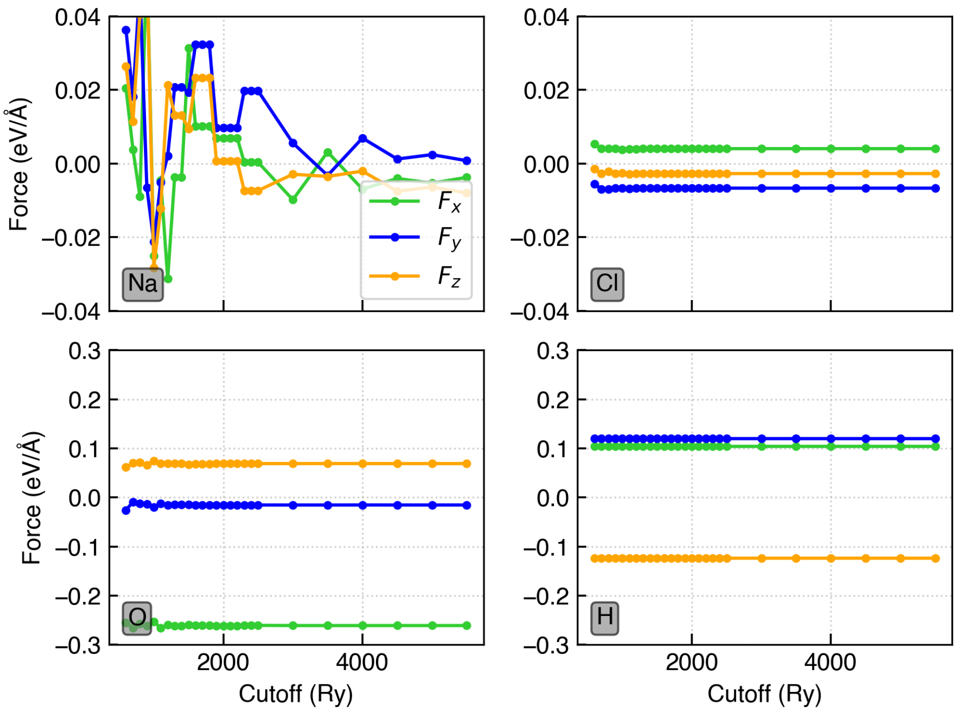

Figure S1: Convergence plot of the force components vs. plane wave cutoff for a random structure of the aqueous NaCl system for all four atom types (Na, Cl, O and H) using a relative plane wave cutoff of 80 Ry.

Figure S2 shows that the plane wave cutoff converges around 1000 Ry using the GAPW method for all force components on a Na^+ cation. To ensure that all convergence issues are eliminated, we selected a plane wave cutoff of 2000 Ry.

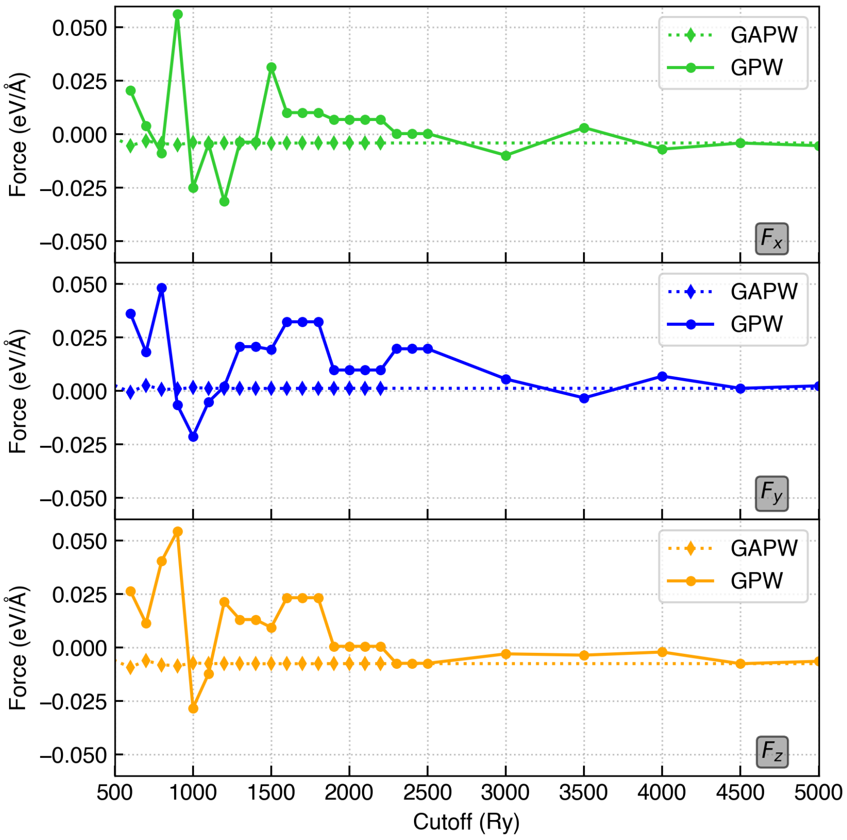

Figure S2: Convergence plot of the force components on Na vs. plane wave cutoff for a random structure of the aqueous NaCl system for GPW and GAPW method using a relative plane wave cutoff of 80 Ry.

Moreover, we tested the convergence of the relative plane wave cutoff for the GAPW method. The absolute plane wave cutoff was set to 2000 Ry. The plot is shown in Fig. S3.

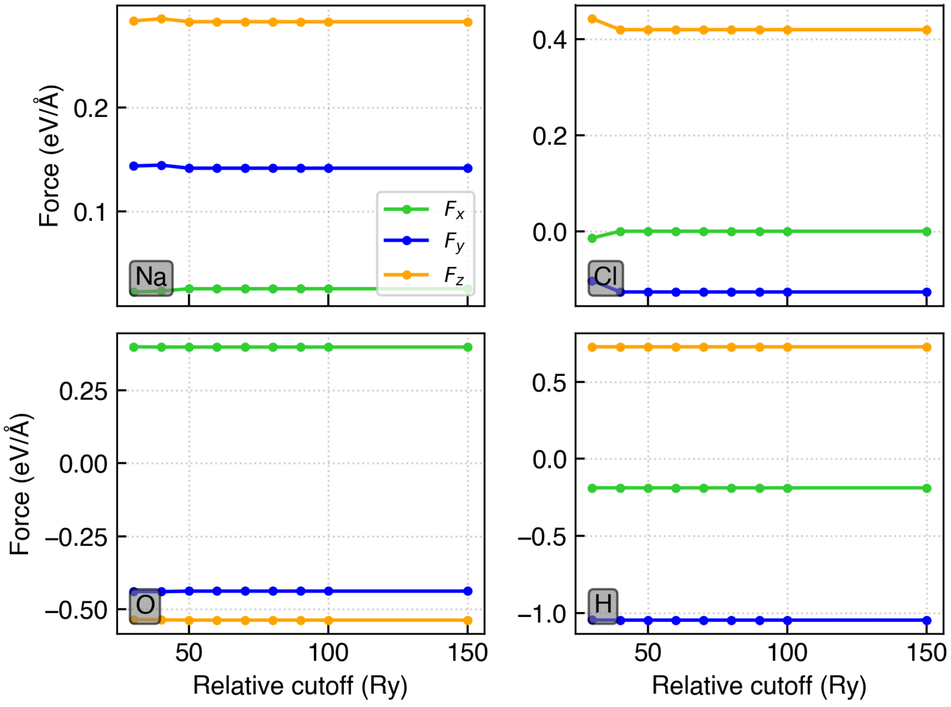

Figure S3: Convergence plot of the force components vs. relative plane wave cutoff for a random structure of the aqueous NaCl system for all four atom types (Na, Cl, O and H) using an absolute plane wave cutoff of 2000 Ry together with the GAPW method.

All convergence tests were conducted using five multigrids in accordance with the reference data.

1.5 Training data augmentation to probe long-range effects

For the generation of additional configurations used in the long-range interaction study, larger simulation boxes containing one or two sodium chloride ion pairs and 146 or 149 water molecules, respectively, were considered. Initial configurations were generated using the program package `packmol`[S1]. The systems were equilibrated using classical molecular dynamics simulations performed with the program package GROMACS[S2] employing the non-polarizable OPLS-AA force field[S3] in combination with the SPC water model.

The simulations were carried out under periodic boundary conditions. Following an initial energy minimization, the systems were equilibrated in the NPT ensemble at 300 K and 1 bar

to relax the simulation box size. Subsequently, a second NPT simulation was performed to obtain an average equilibrium box volume. The resulting side lengths of the cubic simulation boxes are shown in Table S2 below.

Table S2: Set up of the simulation boxes used for the classical MD-based structure generation for the long-range interaction study under periodic boundary conditions.

Number of ion pairs	Box side length [Å]
1 NaCl	16.742
2 NaCl	16.674

The box dimensions were then fixed to this average value, and the systems were further equilibrated in the NVT ensemble at 300 K. A final production run of 20 ns was carried out under NVT conditions.

From these simulations, 100 configurations were evenly extracted, spanning the full range of ion–ion separations, for each concentration (one and two ion pairs) and recomputed at the DFT level using the same electronic-structure setup as described in Section 1.3 to obtain reference energies and forces for training. After post-processing and removing non-converged structures, the data set augmentation comprised 90 structures with one ion pair and 84 structures with two ion pairs.

2 Training of the MLPs

Training was conducted using MACE[S20] version 0.3.5 and using version 0.3.14 for the long-range models. The maximum correlation order in the atomic cluster expansion was $\nu = 3$. During training, the force weight was $\lambda_F = 1000$, and the energy weight was $\lambda_E = 10$. Stochastic weight averaging (SWA) was employed in the last 25% of epochs, increasing the energy weight by 20% with exponential moving averaging (EMA) used otherwise. All models were trained for 600 epochs. A batch size of two was used for training, and four for validation. If not explicitly mentioned, the default settings of MACE version 0.3.5 were used.

3 Numerical validation of MLPs

3.1 Error metrics

Numerical errors were computed using the root-mean-squared error (RMSE) for the energies:

$$\text{RMSE } E = \sqrt{\frac{1}{N}\sum_{i=1}^{N}\left(E_i^{\text{MACE}} - E_i^{\text{DFT}}\right)^2} \tag{1}$$

where E_i^{DFT} is the ground-truth energy given by the reference dataset and E_i^{MACE} the energy predicted by the MACE model for the i-th configuration. For the forces, we employed the magnitude-based force RMSE (RMSE_{mag}) as defined in Ref.[S21]:

$$\text{RMSE}_{\text{mag}}\ |F| = \sqrt{\frac{1}{N}\sum_{i=1}^{N}\left\|\mathbf{F}_i^{\text{MACE}} - \mathbf{F}_i^{\text{DFT}}\right\|^2} \tag{2}$$

Here, the index i runs over all atoms in all configurations. $\mathbf{F}_i^{\text{DFT}}$ and $\mathbf{F}_i^{\text{MACE}}$ denote the ground-truth and predicted force vectors, respectively.

For better comparability to other studies, we also include the component-wise RMSE for forces defined as:

$$\text{RMSE } F_i = \sqrt{\frac{1}{3N}\sum_{i=1}^{N}\sum_{j=1}^{3}\left(F_{ij}^{\text{MACE}} - F_{ij}^{\text{DFT}}\right)^2}. \tag{3}$$

The results are shown in Figure S4.

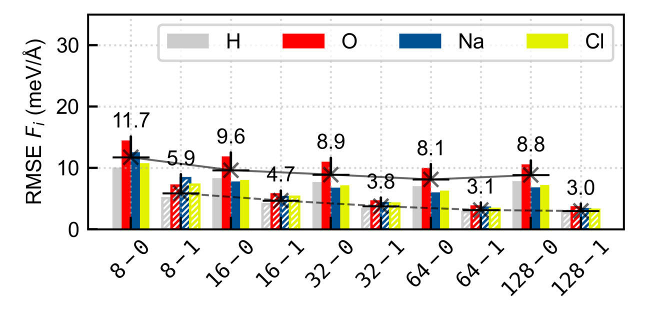

Figure S4: Componentwise force RMSEs according to Eq. 3 of trained MLPs with varying combinations of k and L_{max}, where we employ the same nomenclature as in Ref. S22: k–L_{max}.

Additionally, we compute a relative RMSE (RRMSE) for the energies according to

$$\text{RRMSE } E = \left| \frac{\text{RMSE}}{\langle E^{\text{DFT}} \rangle} \right| \tag{4}$$

where the RMSE is normalized by the mean of the ground-truth energies $\langle E^{\text{DFT}} \rangle$.

For the component-wise force RRMSE, we normalized via the mean absolute values of the force vector components:

$$\text{RRMSE } F_i = \frac{\text{RMSE } F_i}{\frac{1}{3N} \sum_{i=1}^{N} \sum_{j=1}^{3} |F_{ij}^{\text{DFT}}|}. \tag{5}$$

And finally, for the relative RMSE_{mag} we also normalize by the mean magnitude of the force vector of the respective element:

$$\text{RRMSE}_{\text{mag}} |F| = \frac{\text{RMSE}_{\text{mag}} |F|}{\frac{1}{N} \sum_{i=1}^{N} \|\mathbf{F}_i^{\text{DFT}}\|}. \tag{6}$$

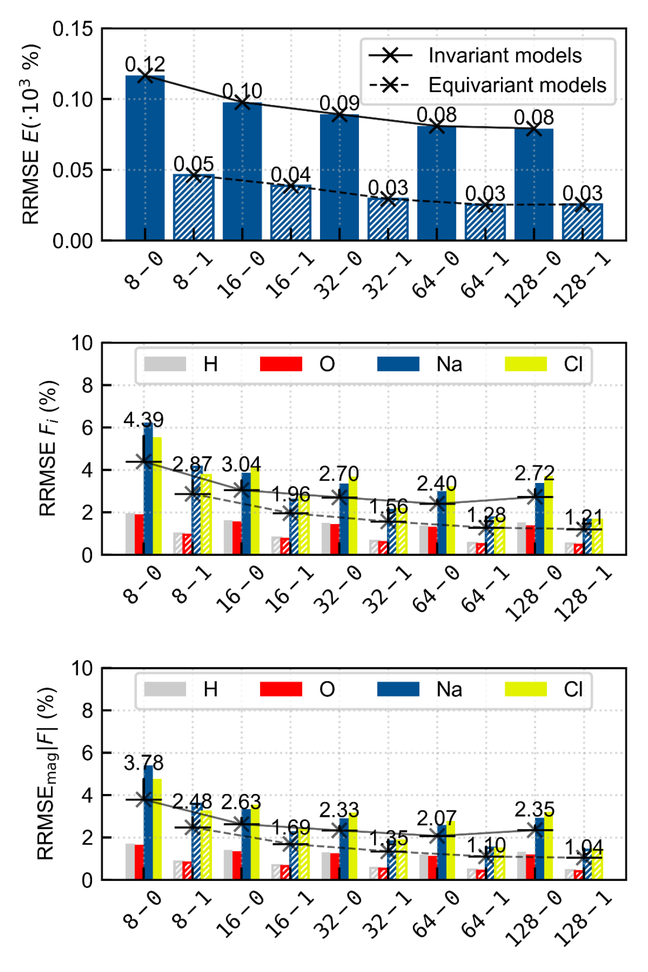

Figure S5: Relative errors of trained MLPs with varying combinations of k and $L_{\max}$, where we employ the same nomenclature as in Ref.[S22]: $k - L_{\max}$. The upper panel shows the energy errors according to Eq. 4. The center panel shows the element-wise RRMSE of the component-wise force RMSE and the bottom panel the element-wise RRMSE$_{\text{mag}}$.

3.2 Further numerical validation results of all MLPs

Here, we report the errors in predicted energies and forces relative to the test dataset, including histograms of energy errors, energy correlation plots, and element-wise force correlation

8-0

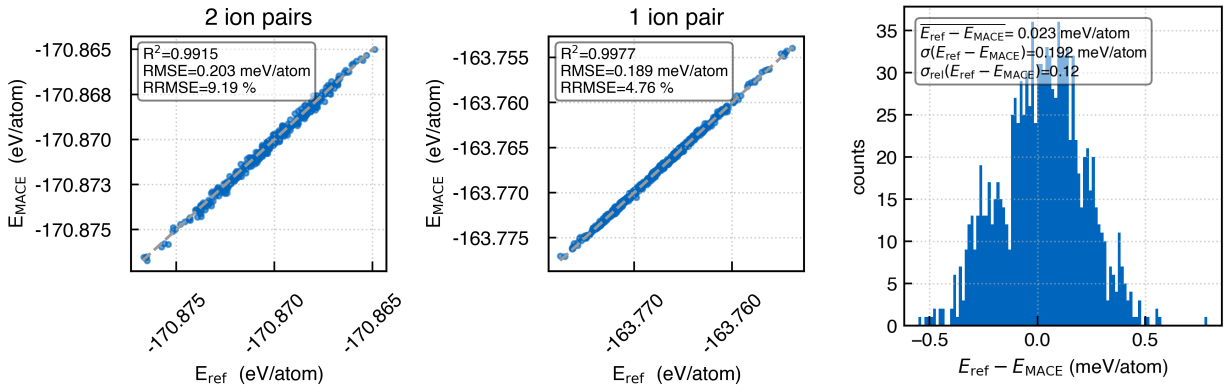

Figure S6: Energy errors of the 8-0 model on the test dataset. The left panel shows parity plots of E_{MACE} versus the reference DFT energies E_{ref}, normalized per atom. Configurations with one and two ion pairs are shown separately to reflect the discrete energy levels in the test set. The right panel shows a histogram of the corresponding energy differences.

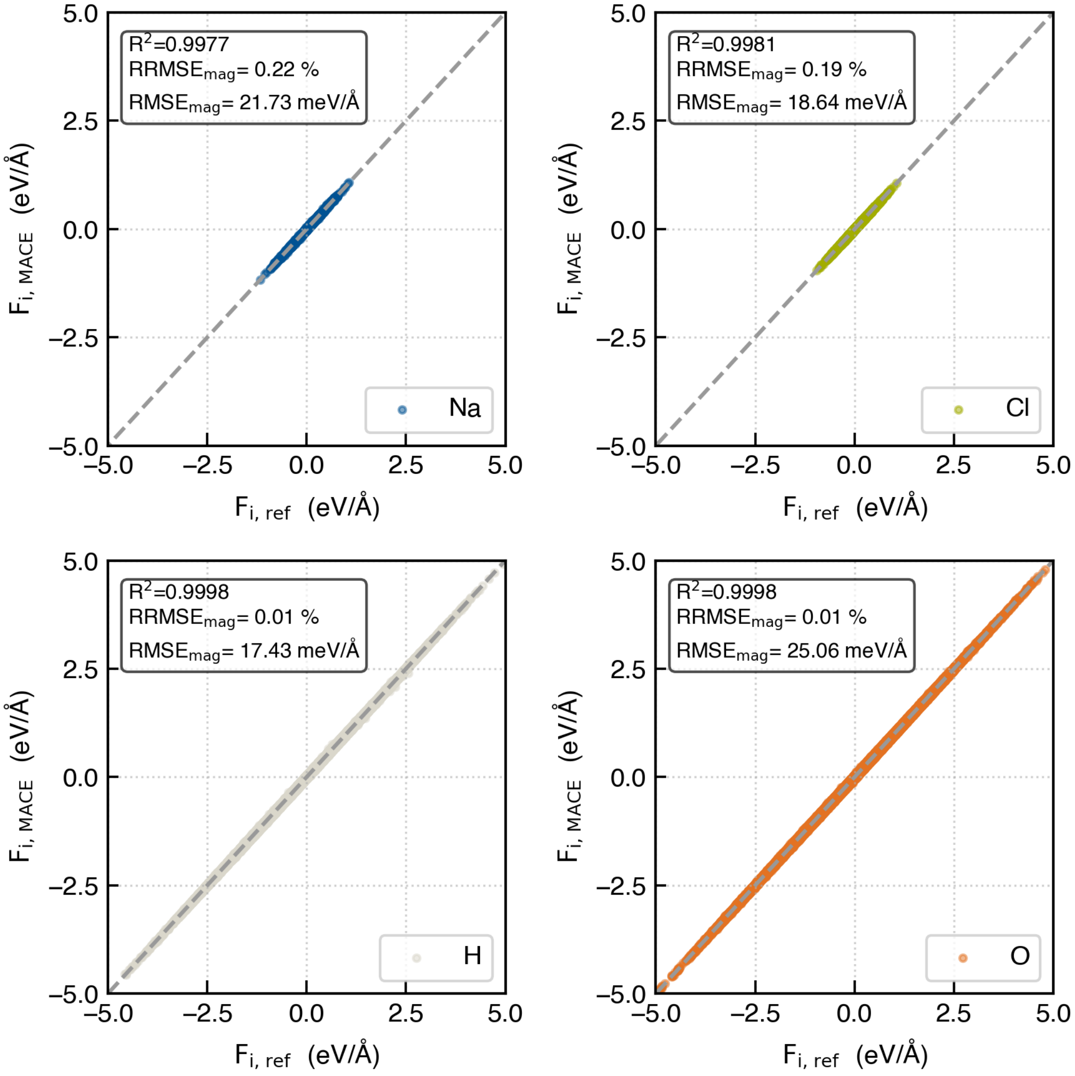

Figure S7: Element-wise force correlation between the components predicted by the 8-0 model $F_{i,\mathrm{MACE}}$ and the reference data $F_{i,\mathrm{ref}}$

8-1

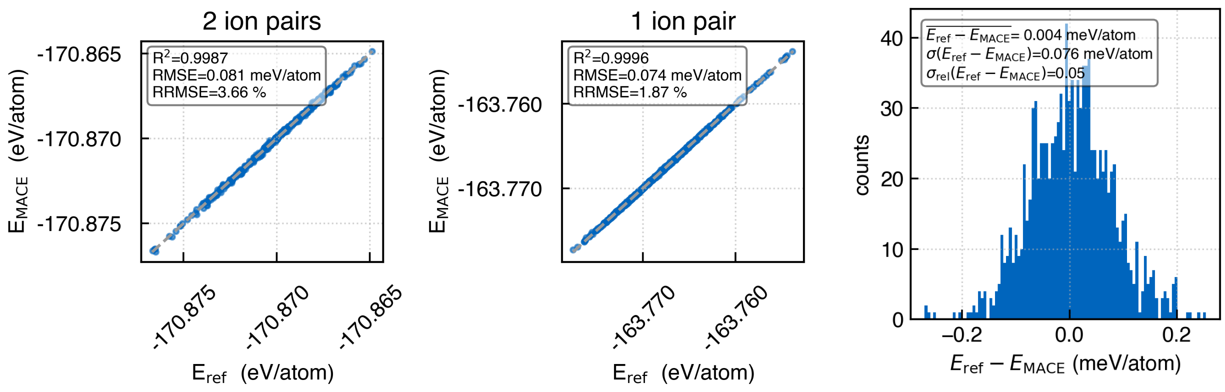

Figure S8: Energy errors of the 8-1 model on the test dataset. The left panel shows parity plots of E_{MACE} versus the reference DFT energies E_{ref}, normalized per atom. Configurations with one and two ion pairs are shown separately to reflect the discrete energy levels in the test set. The right panel shows a histogram of the corresponding energy differences.

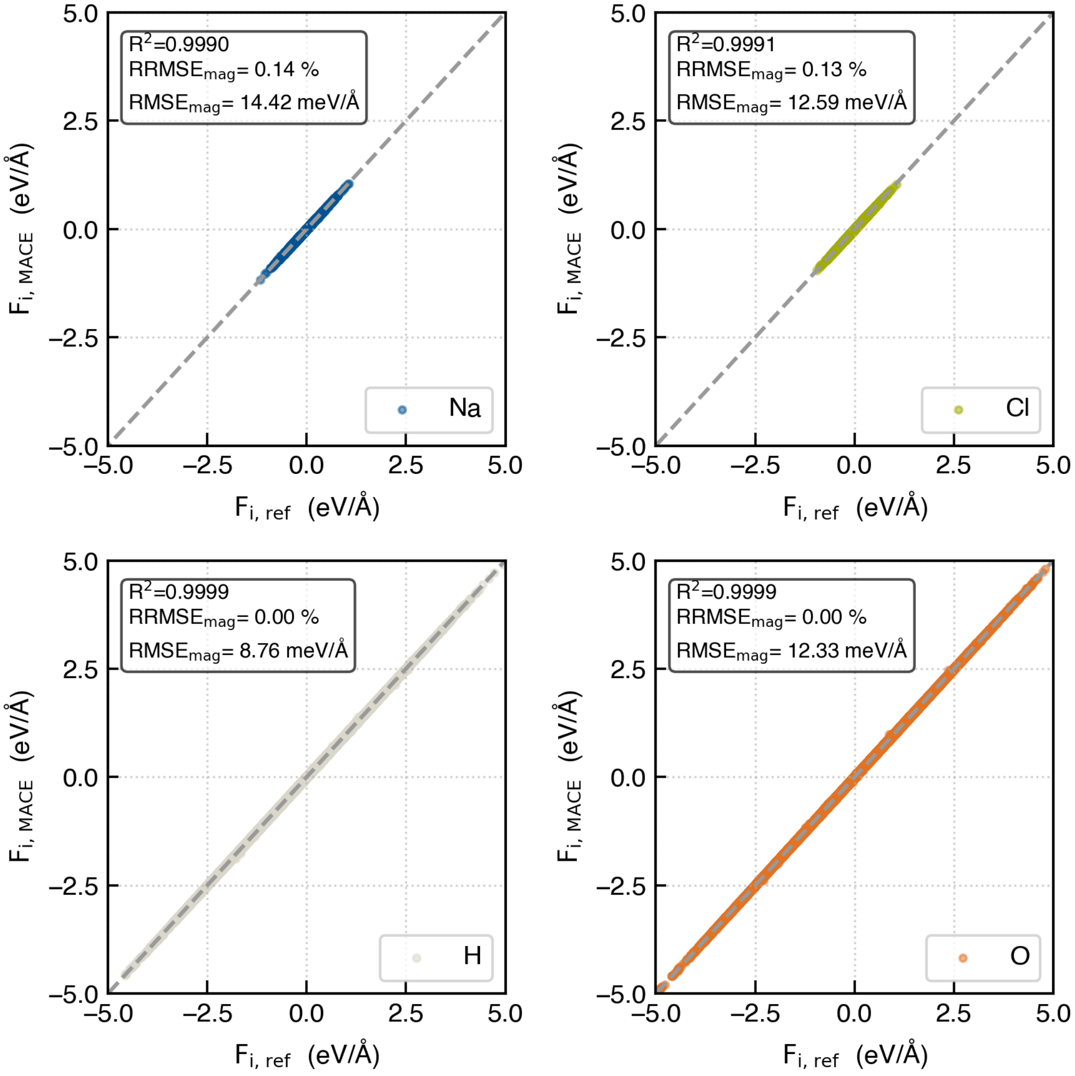

Figure S9: Element-wise force correlation between the components predicted by the `8-1` model $F_{i,\mathrm{MACE}}$ and the reference data $F_{i,\mathrm{ref}}$

16-0

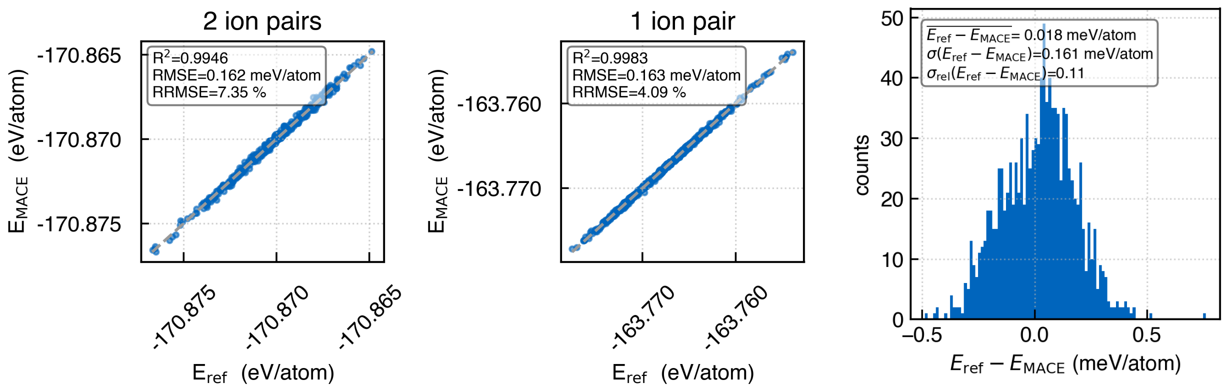

Figure S10: Energy errors of the 16-0 model on the test dataset. The left panel shows parity plots of E_{MACE} versus the reference DFT energies E_{ref}, normalized per atom. Configurations with one and two ion pairs are shown separately to reflect the discrete energy levels in the test set. The right panel shows a histogram of the corresponding energy differences.

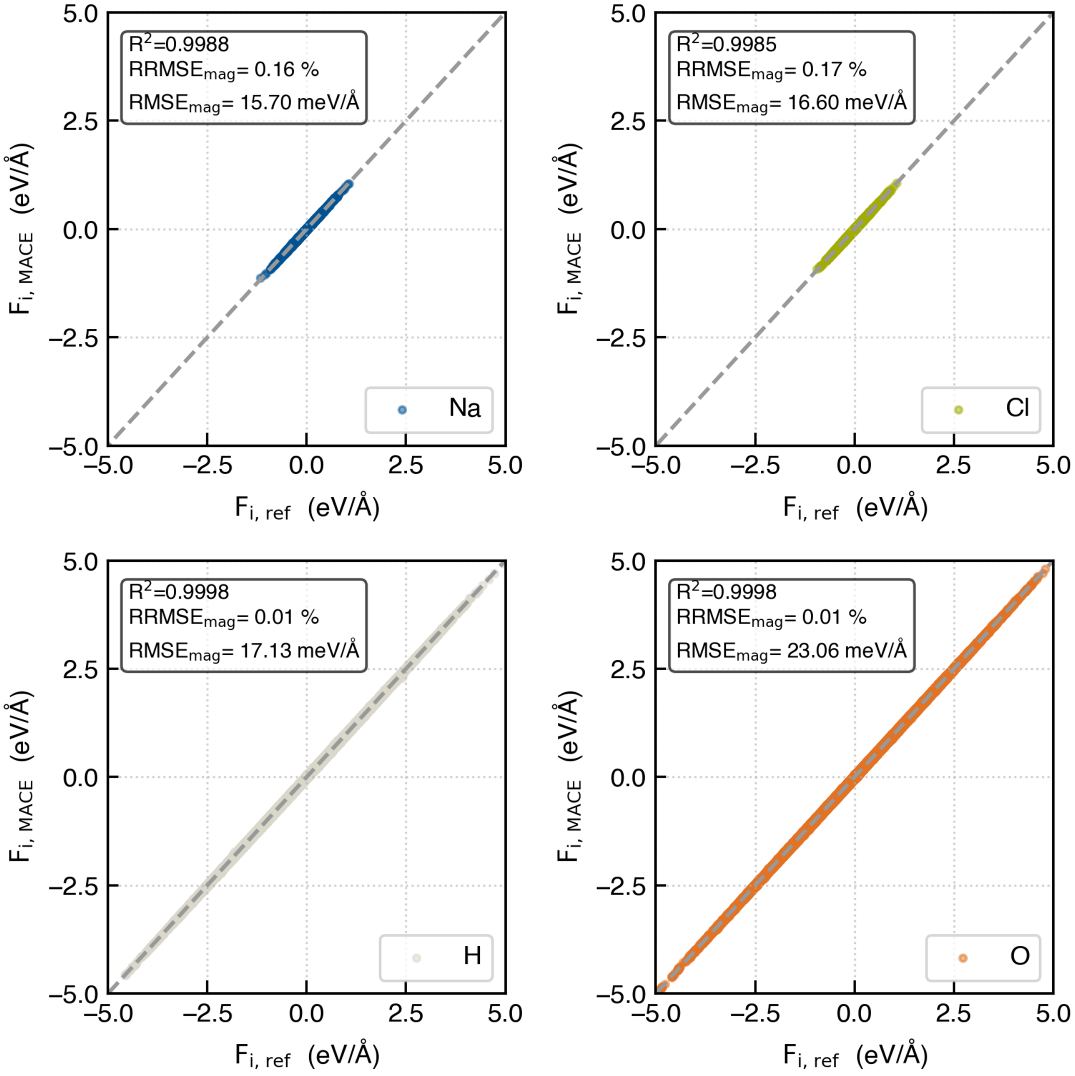

Figure S11: Element-wise force correlation between the components predicted by the `16-0` model $F_{i,\mathrm{MACE}}$ and the reference data $F_{i,\mathrm{ref}}$

16-1

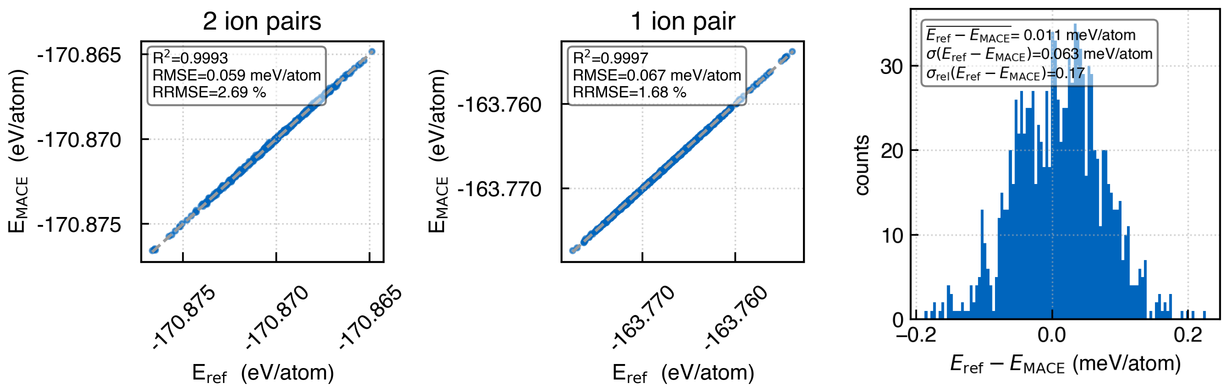

Figure S12: Energy errors of the `16-1` model on the test dataset.The left panel shows parity plots of E_{MACE} versus the reference DFT energies E_{ref}, normalized per atom. Configurations with one and two ion pairs are shown separately to reflect the discrete energy levels in the test set. The right panel shows a histogram of the corresponding energy differences.

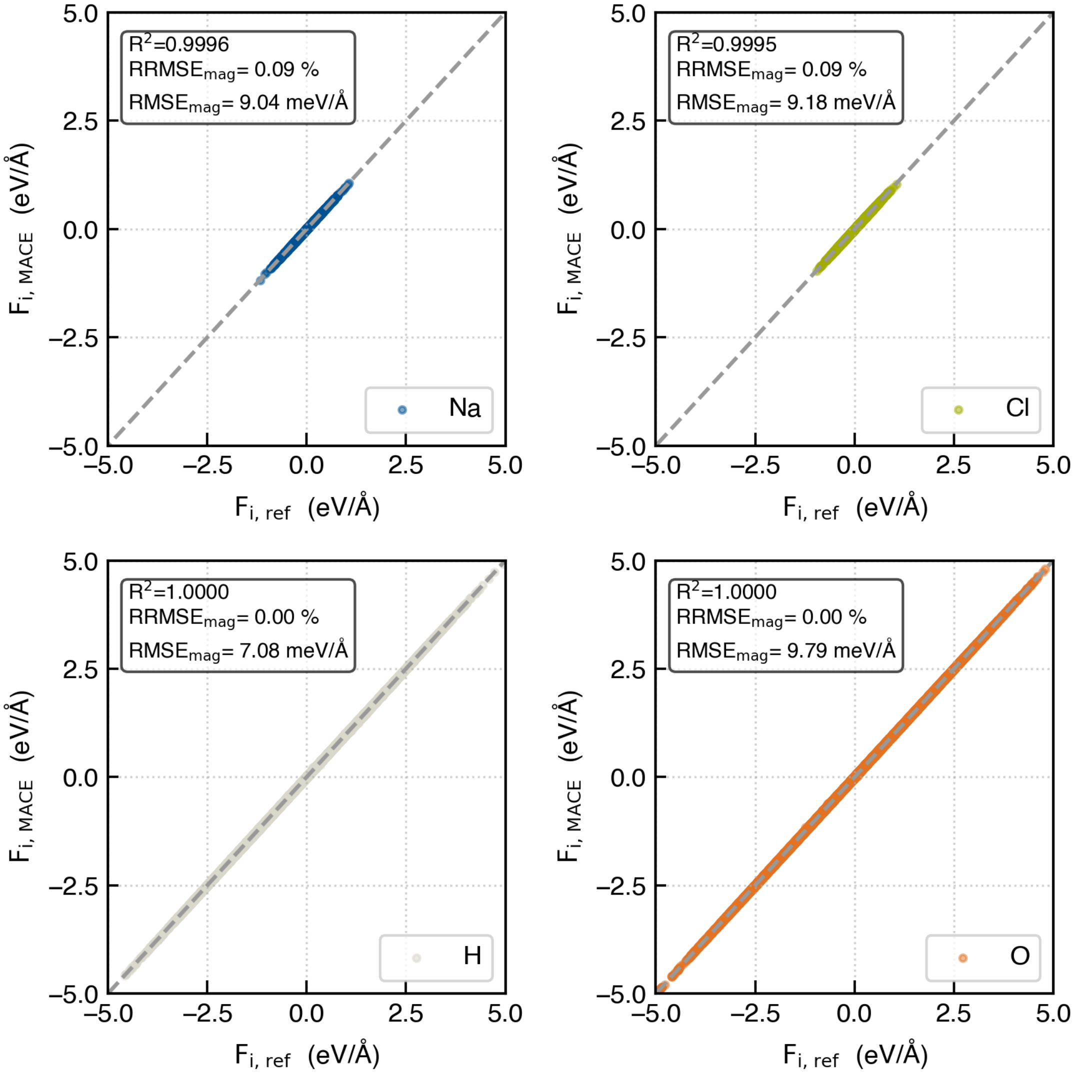

Figure S13: Element-wise force correlation between the components predicted by the `16-1` model $F_{i,\mathrm{MACE}}$ and the reference data $F_{i,\mathrm{ref}}$

32-0

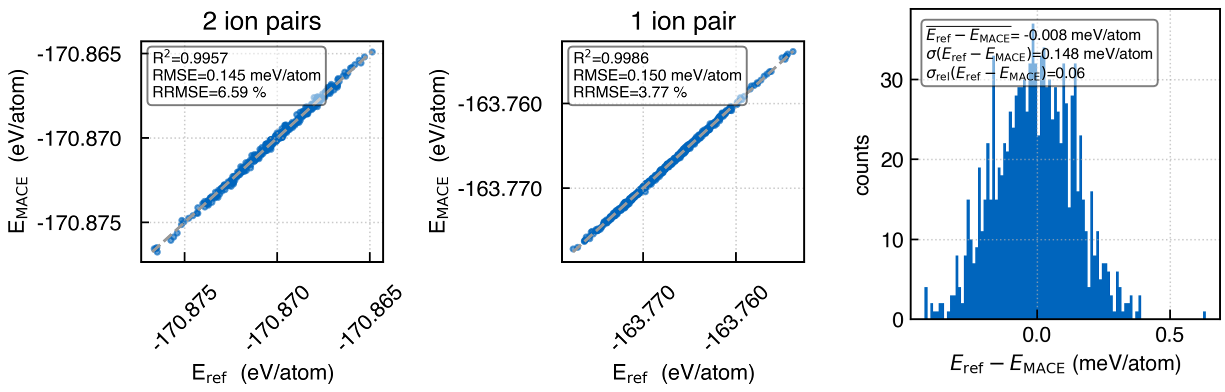

Figure S14: Energy errors of the 32-0 model on the test dataset. The left panel shows parity plots of E_{MACE} versus the reference DFT energies E_{ref}, normalized per atom. Configurations with one and two ion pairs are shown separately to reflect the discrete energy levels in the test set. The right panel shows a histogram of the corresponding energy differences.

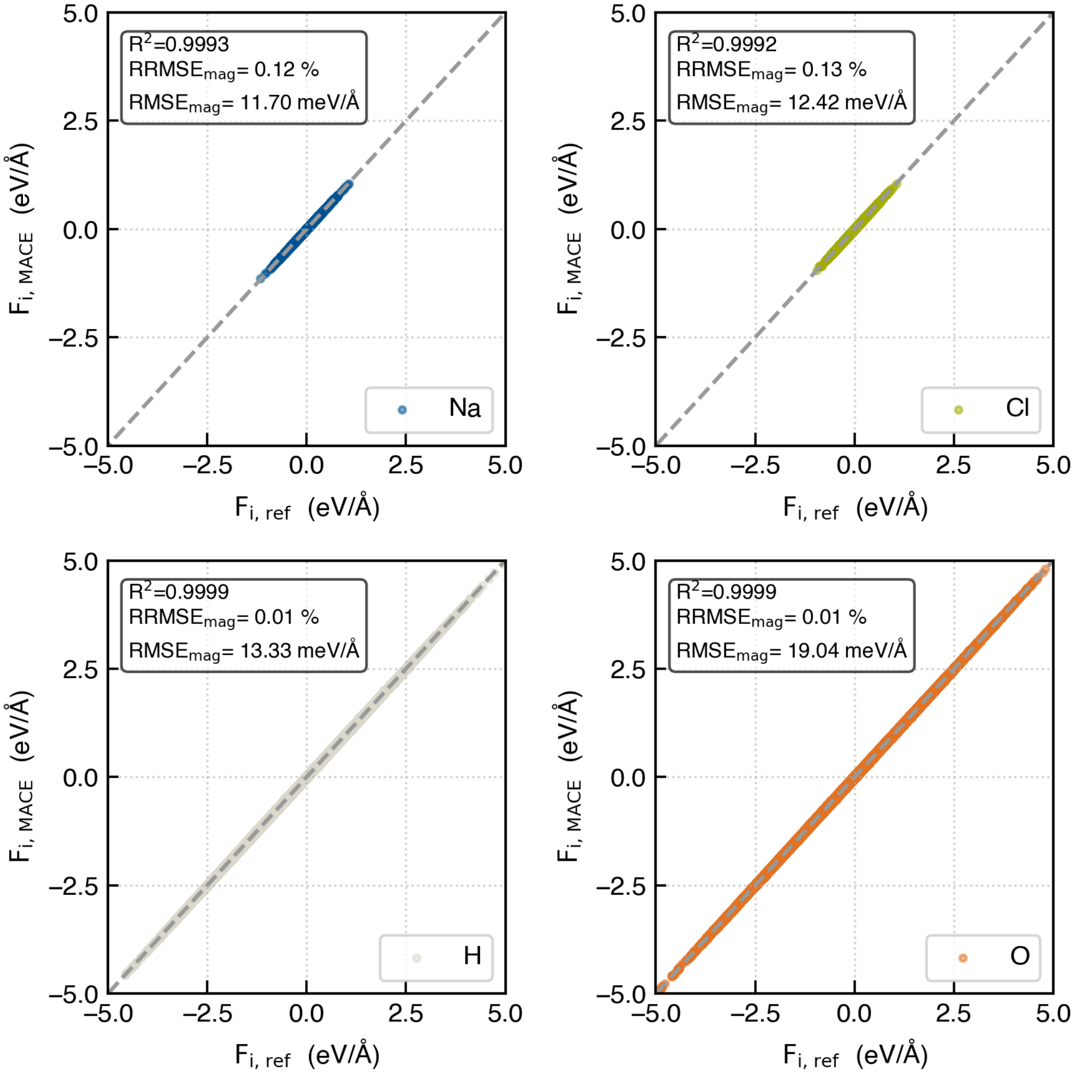

Figure S15: Element-wise force correlation between the components predicted by the 32-0 model $F_{i,\mathrm{MACE}}$ and the reference data $F_{i,\mathrm{ref}}$

32-1

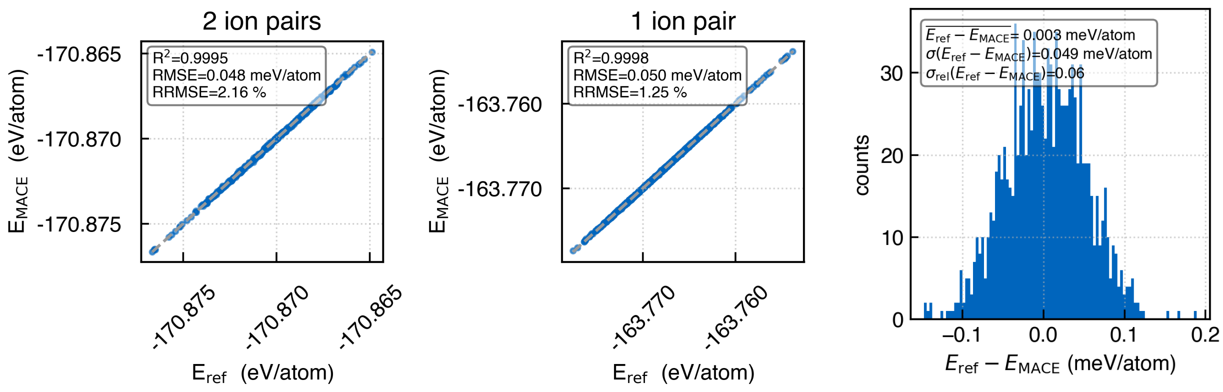

Figure S16: Energy errors of the `32-1` model on the test dataset. The left panel shows parity plots of E_{MACE} versus the reference DFT energies E_{ref}, normalized per atom. Configurations with one and two ion pairs are shown separately to reflect the discrete energy levels in the test set. The right panel shows a histogram of the corresponding energy differences.

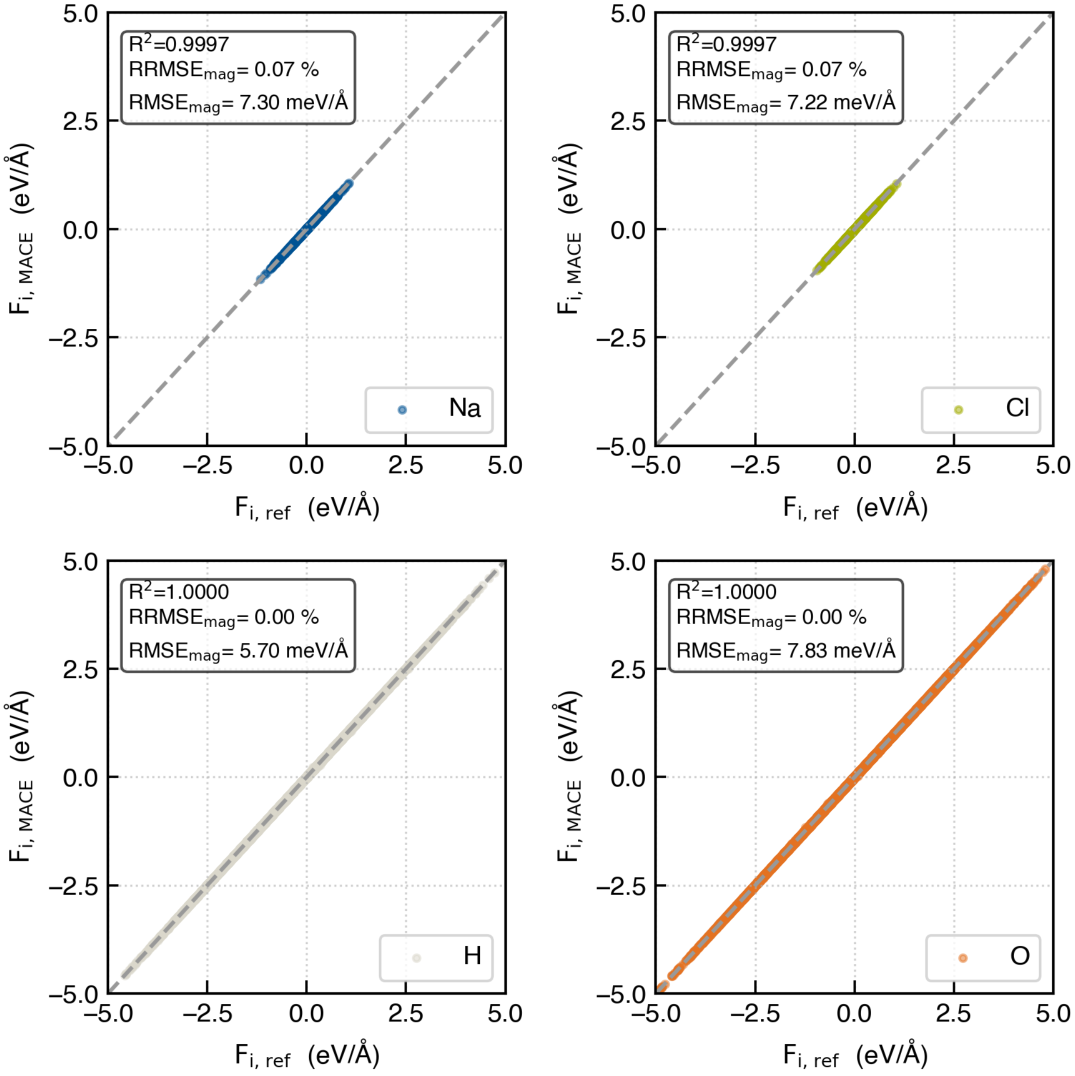

Figure S17: Element-wise force correlation between the components predicted by the 32-1 model $F_{i,\mathrm{MACE}}$ and the reference data $F_{i,\mathrm{ref}}$

64-0

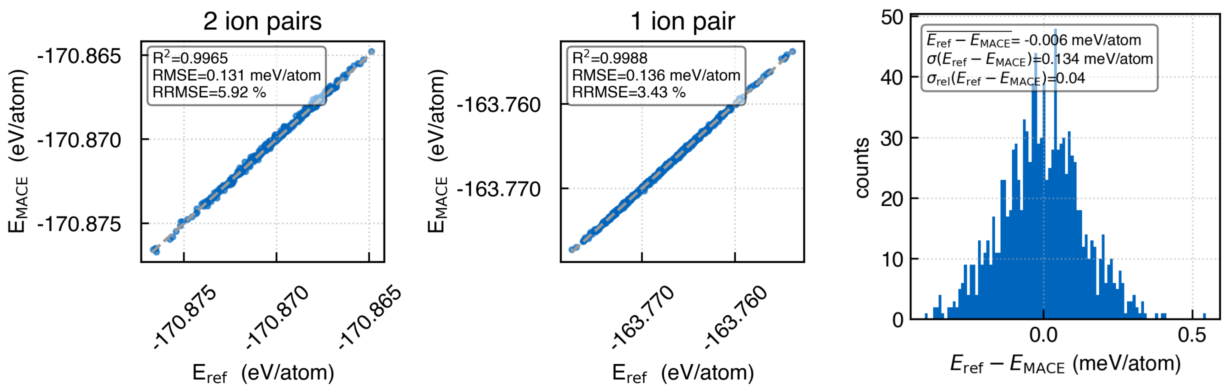

Figure S18: Energy errors of the 64-0 model on the test dataset. The left panel shows parity plots of E_{MACE} versus the reference DFT energies E_{ref}, normalized per atom. Configurations with one and two ion pairs are shown separately to reflect the discrete energy levels in the test set. The right panel shows a histogram of the corresponding energy differences.

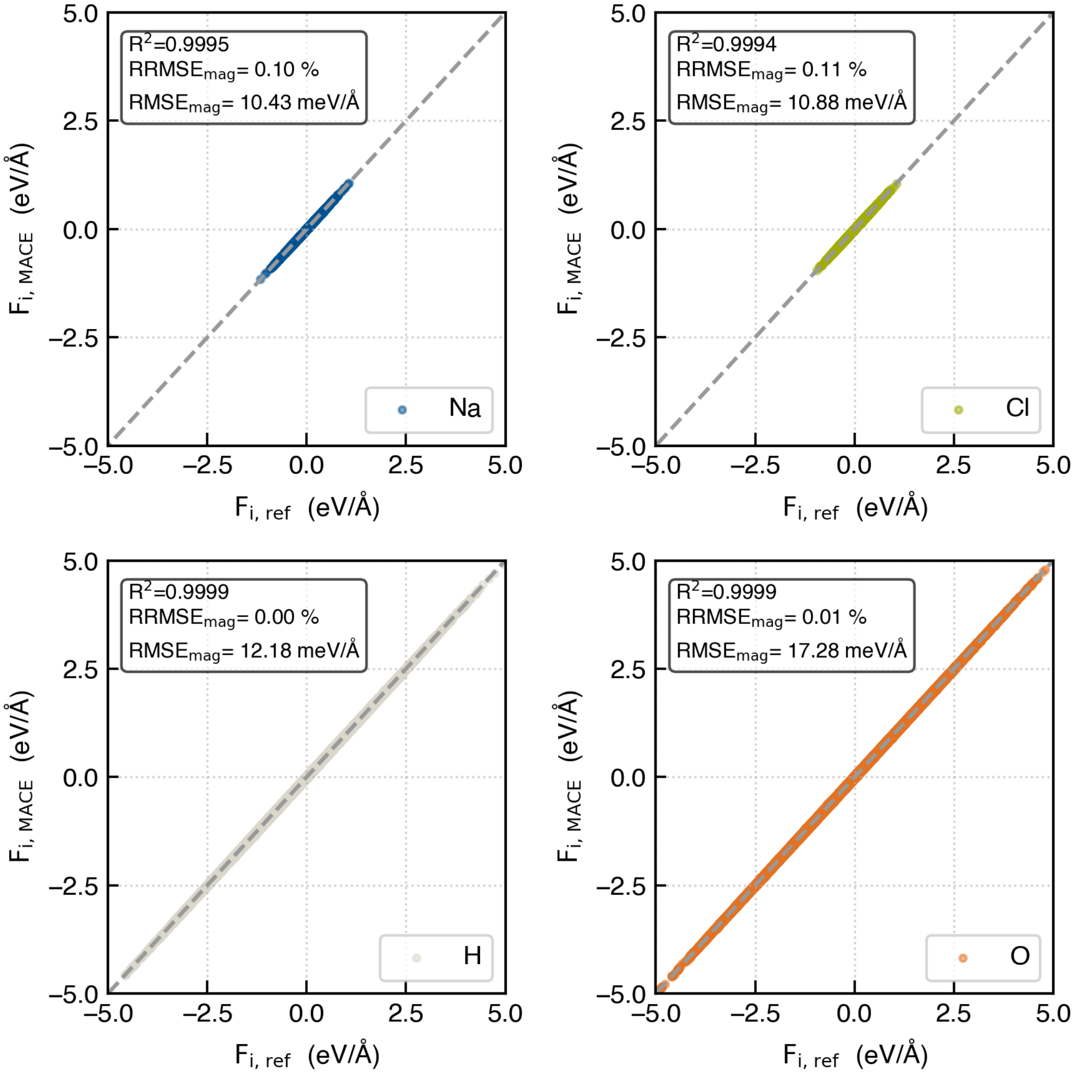

Figure S19: Element-wise force correlation between the components predicted by the `64-0` model $F_{i,\mathrm{MACE}}$ and the reference data $F_{i,\mathrm{ref}}$

64-1

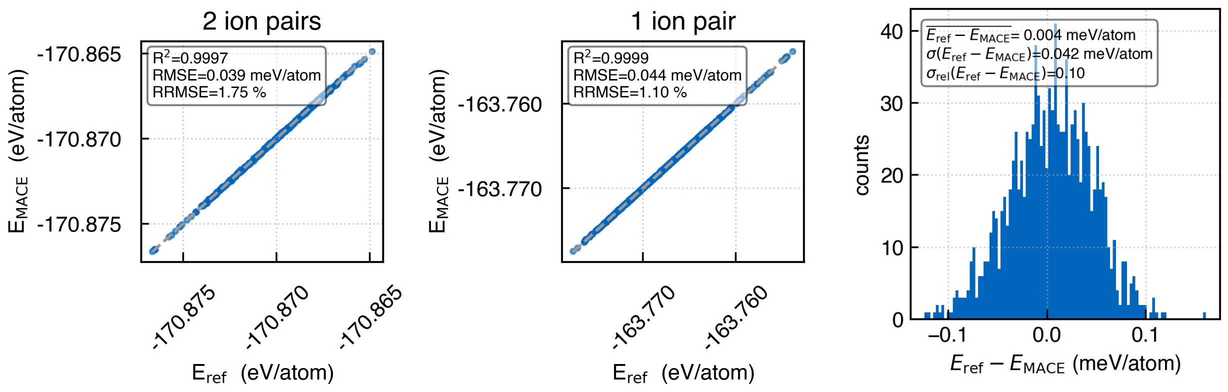

Figure S20: Energy errors of the 64-1 model on the test dataset. The left panel shows parity plots of E_{MACE} versus the reference DFT energies E_{ref}, normalized per atom. Configurations with one and two ion pairs are shown separately to reflect the discrete energy levels in the test set. The right panel shows a histogram of the corresponding energy differences.

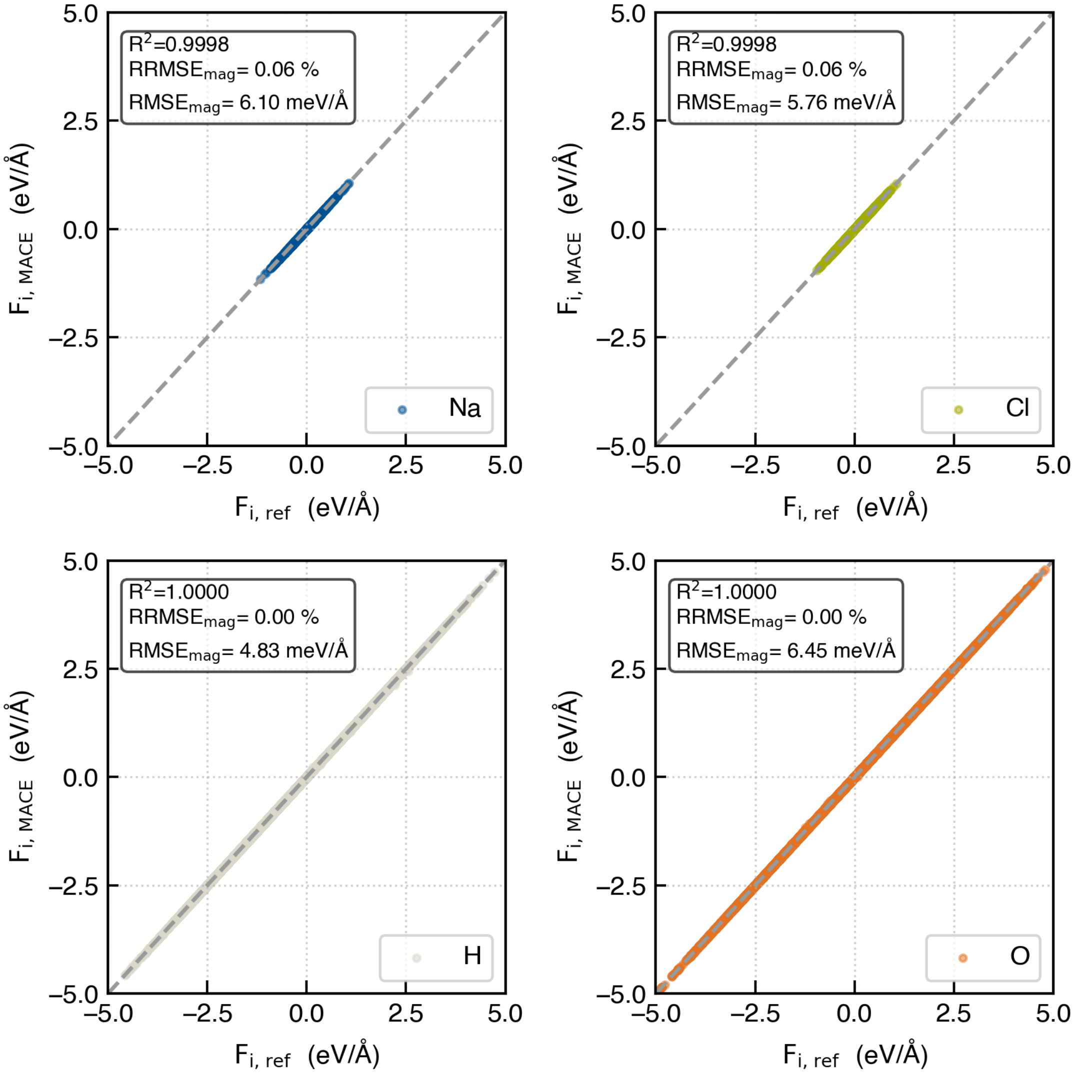

Figure S21: Element-wise force correlation between the components predicted by the `64-1` model $F_{i,\mathrm{MACE}}$ and the reference data $F_{i,\mathrm{ref}}$

128-0

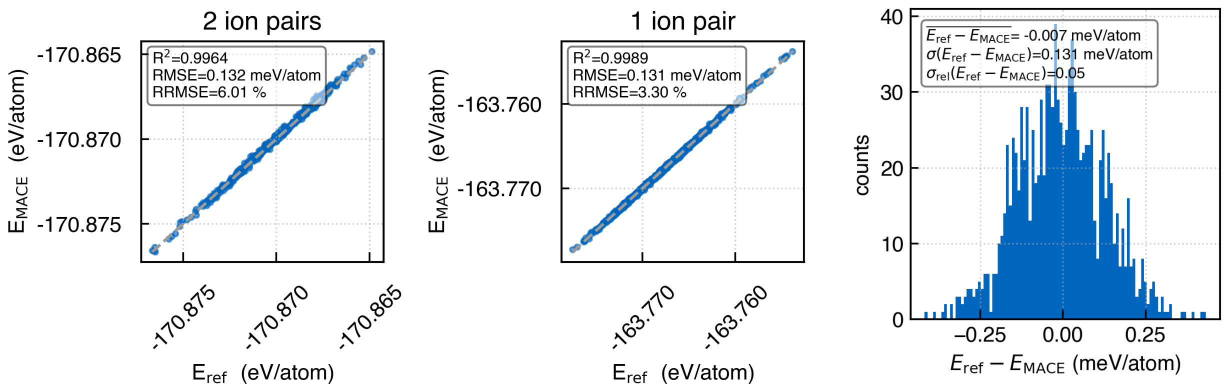

Figure S22: Energy errors of the 128-0 model on the test dataset. The left panel shows parity plots of E_{MACE} versus the reference DFT energies E_{ref}, normalized per atom. Configurations with one and two ion pairs are shown separately to reflect the discrete energy levels in the test set. The right panel shows a histogram of the corresponding energy differences.

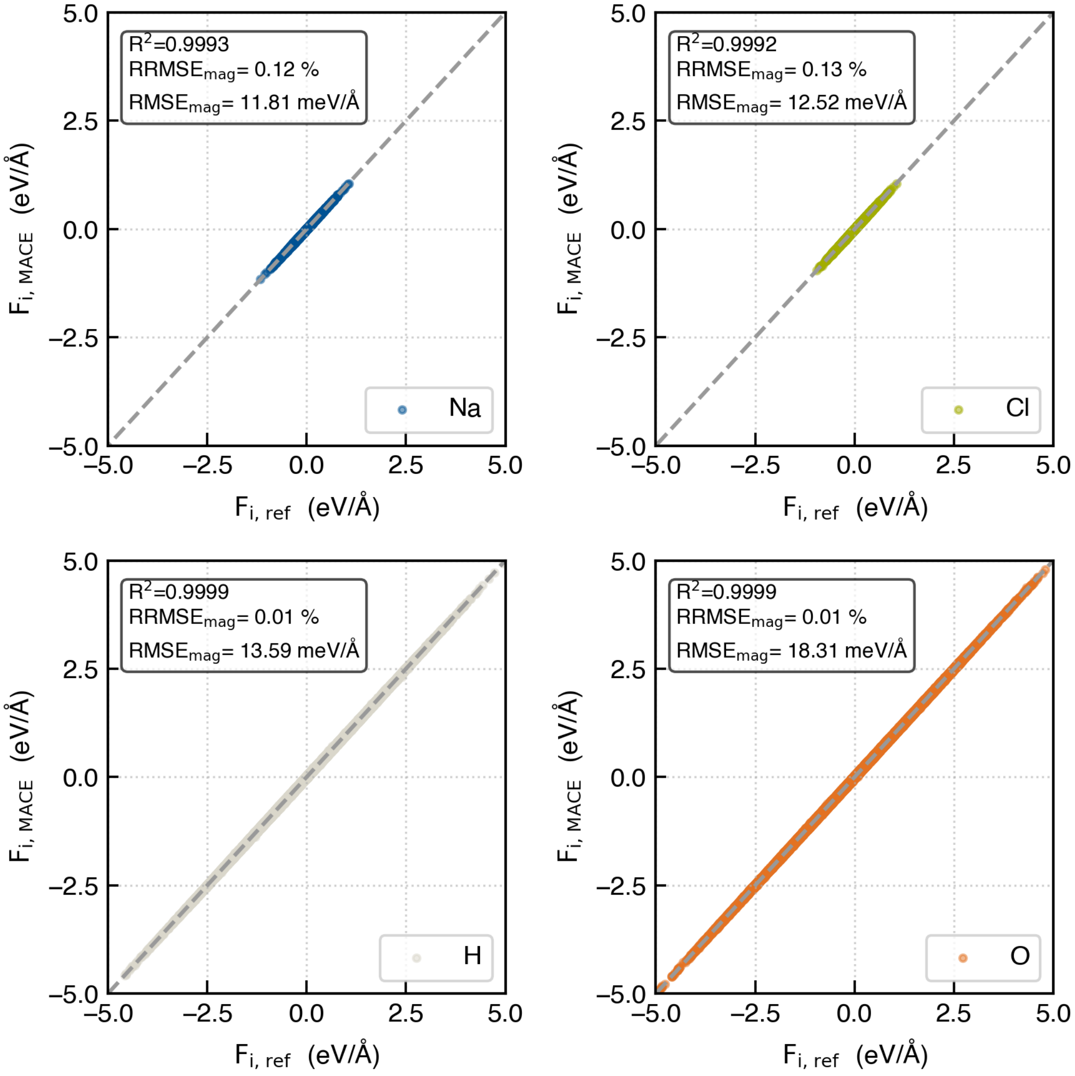

Figure S23: Element-wise force correlation between the components predicted by the `128-0` model $F_{i,\text{MACE}}$ and the reference data $F_{i,\text{ref}}$

128-1

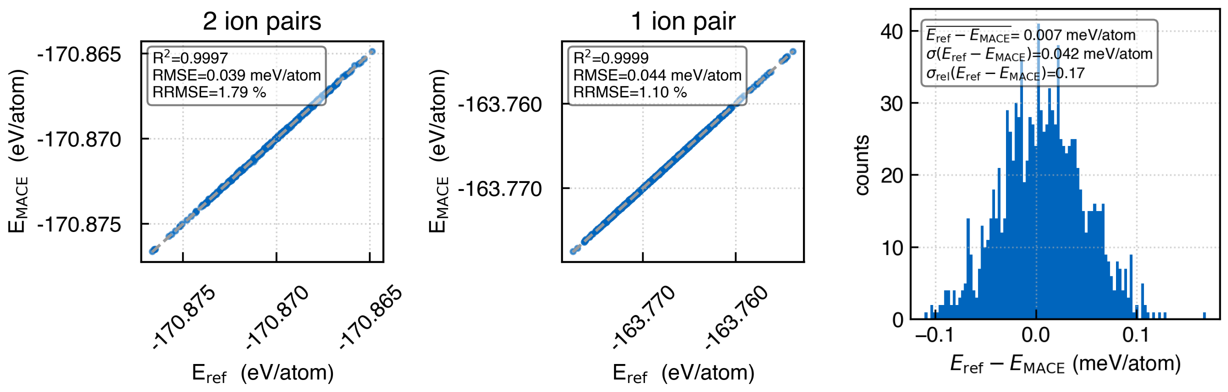

Figure S24: Energy errors of the 128-1 model on the test dataset. The left panel shows parity plots of E_{MACE} versus the reference DFT energies E_{ref}, normalized per atom. Configurations with one and two ion pairs are shown separately to reflect the discrete energy levels in the test set. The right panel shows a histogram of the corresponding energy differences.

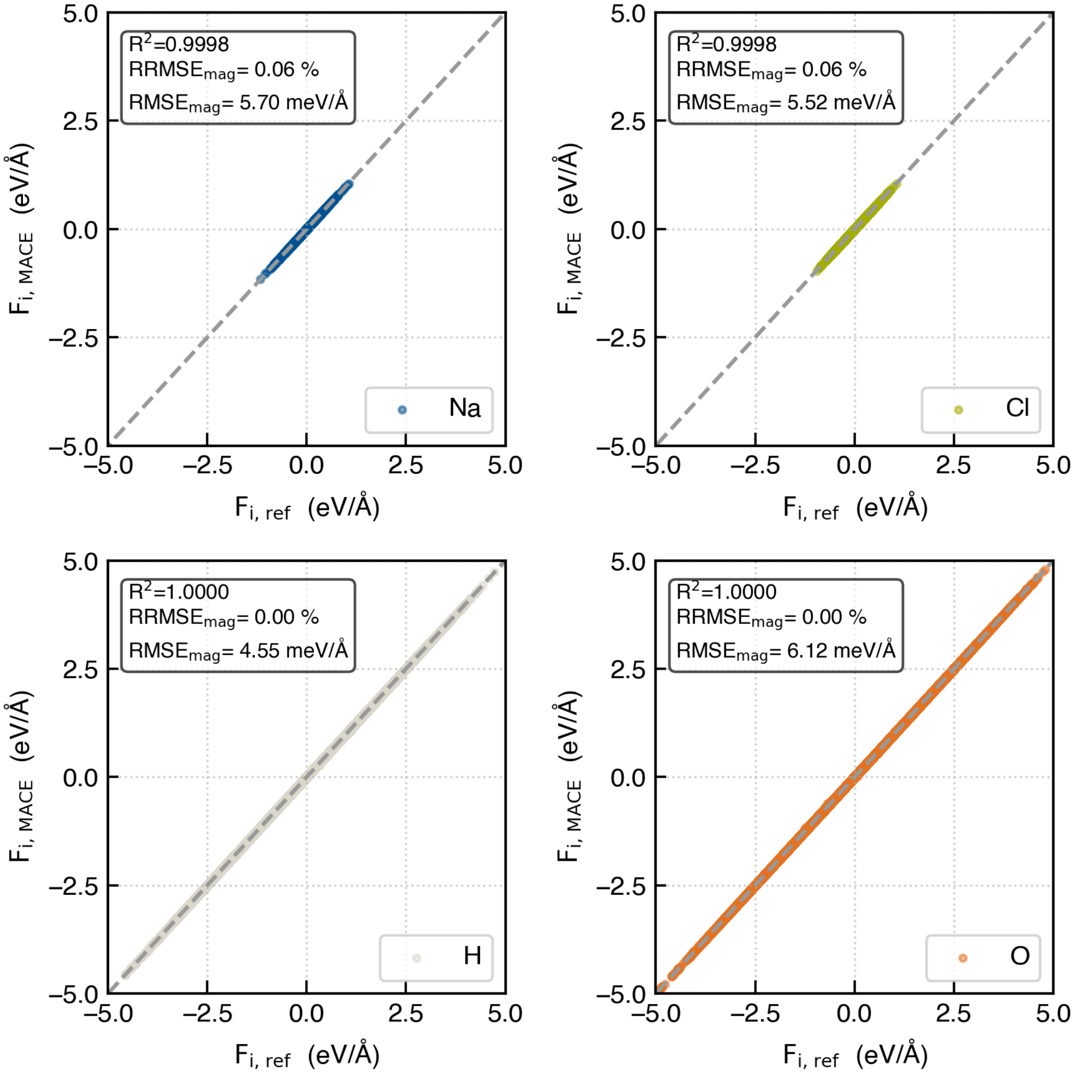

Figure S25: Element-wise force correlation between the components predicted by the `128-1` model $F_{i,\text{MACE}}$ and the reference data $F_{i,\text{ref}}$

32-1 with augmented data set

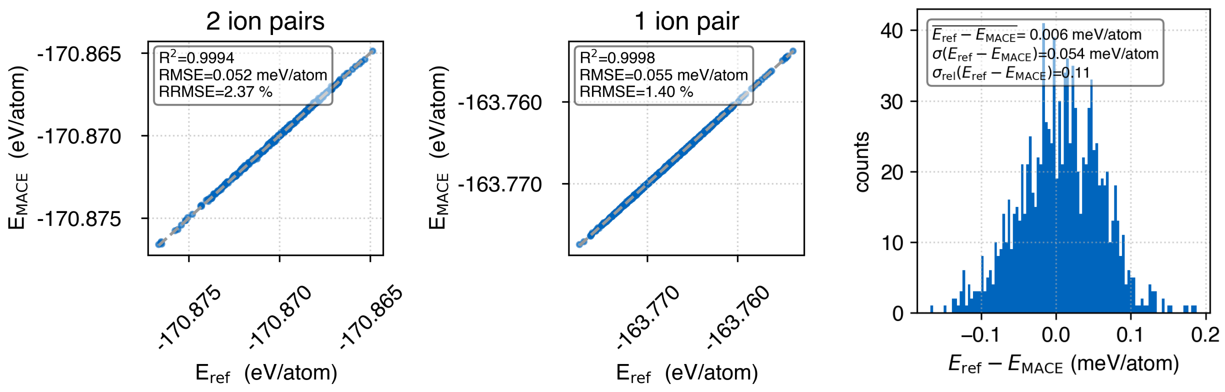

Figure S26: Energy errors of the `32-1` model trained with larger simulation boxes on the test dataset. The left panel shows parity plots of E_{MACE} versus the reference DFT energies E_{ref}, normalized per atom. Configurations with one and two ion pairs are shown separately to reflect the discrete energy levels in the test set. The right panel shows a histogram of the corresponding energy differences.

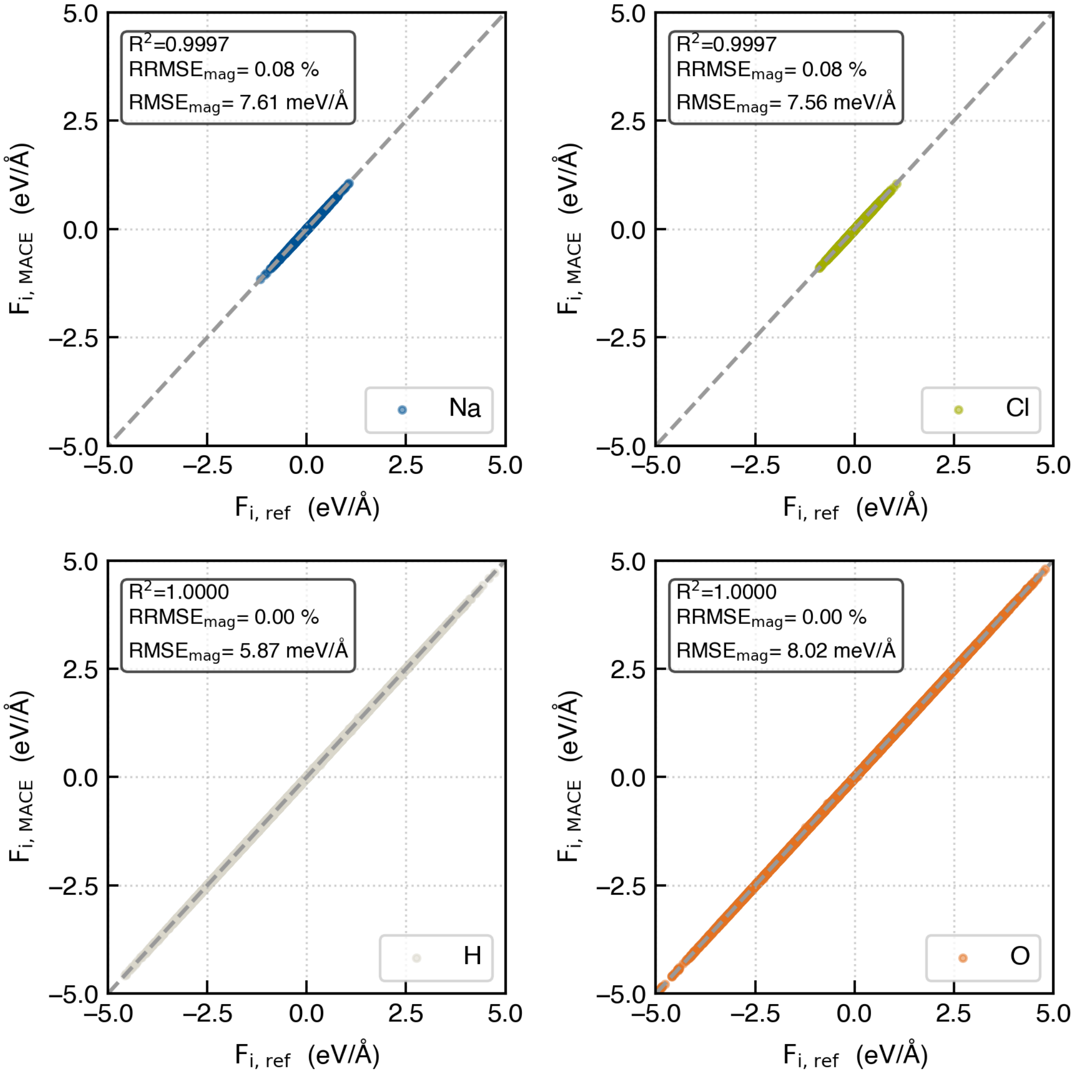

Figure S27: Element-wise force correlation between the components predicted by the 32-1 model trained with larger simulation boxes $F_{i,\mathrm{MACE}}$ and the reference data $F_{i,\mathrm{ref}}$

32-1 with augmented data set and longe-range correction

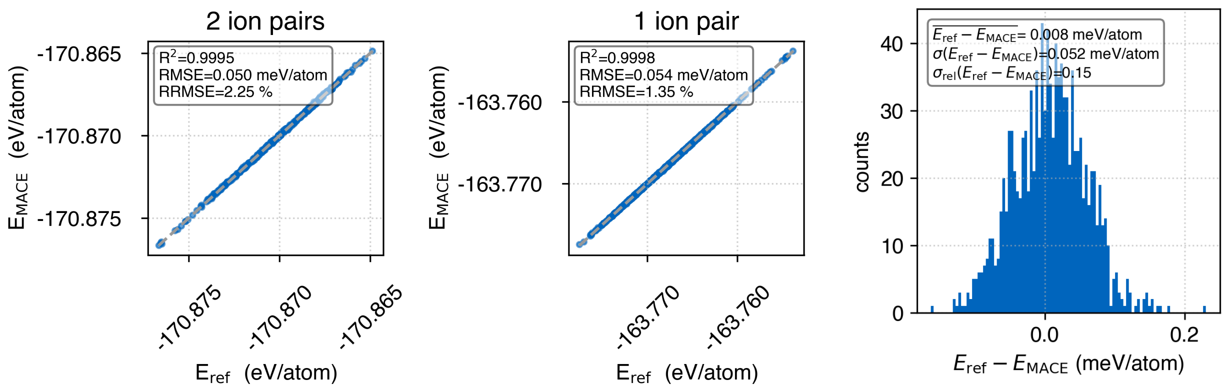

Figure S28: Energy errors of the 32-1 LR-model trained with larger simulation boxes on the test dataset. The left panel shows parity plots of E_{MACE} versus the reference DFT energies E_{ref}, normalized per atom. Configurations with one and two ion pairs are shown separately to reflect the discrete energy levels in the test set. The right panel shows a histogram of the corresponding energy differences.

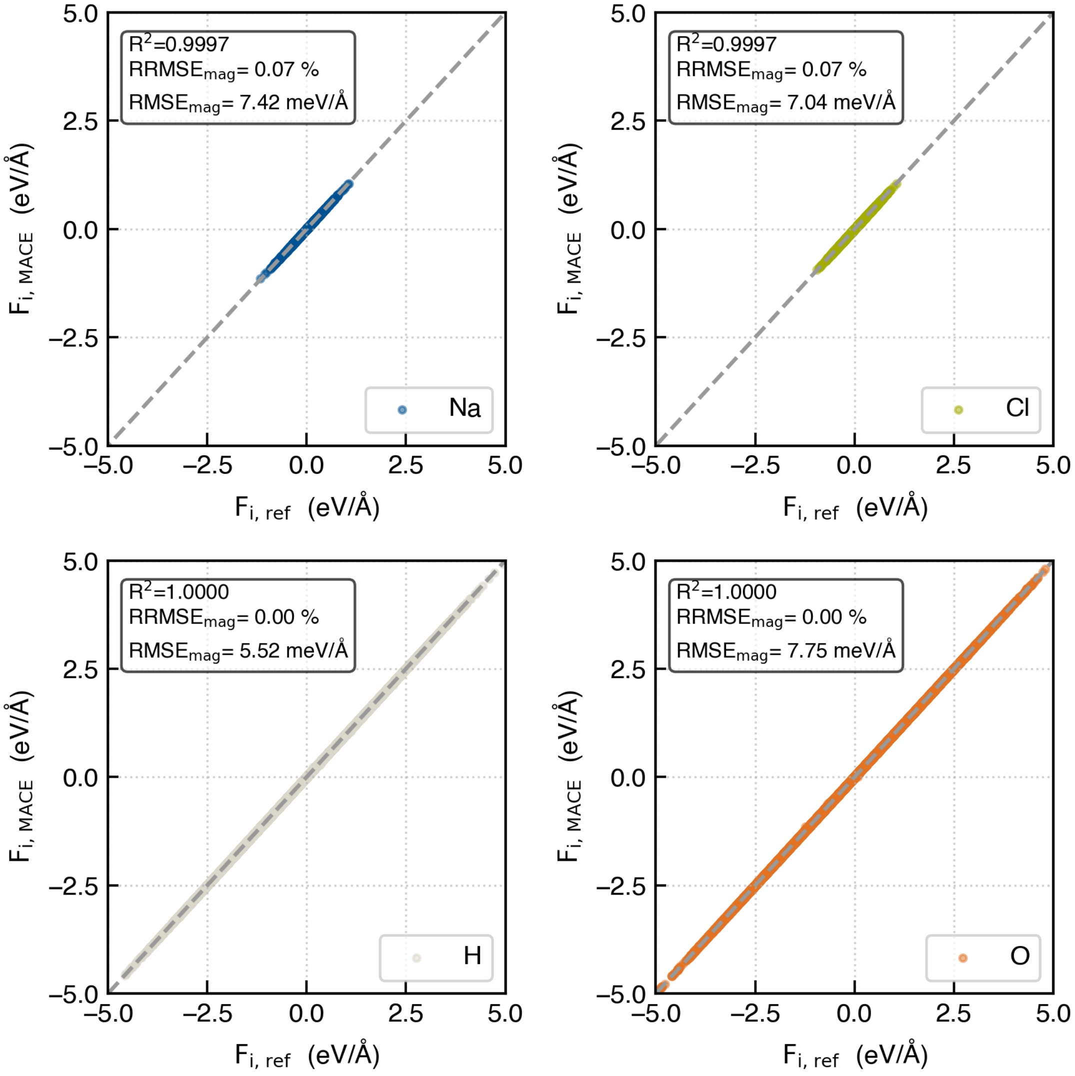

Figure S29: Element-wise force correlation between the components predicted by the 32-1 LR-model trained with larger simulation boxes $F_{i,\mathrm{MACE}}$ and the reference data $F_{i,\mathrm{ref}}$

4 Further information on and results of the MLP simulations

4.1 Calculation of the PMF

The potential of mean force was calculated from the pair-wise radial distribution function $g_{\text{Na–Cl}}(r)$ of the Na^+ and Cl^- ion pair according to

$$\text{PMF} = -RT\ln(g_{\text{Na–Cl}}(r)) \tag{7}$$

where T is the temperature, i.e. 300 K. The PMF was averaged over the five 1 ns trajectories for each trained model.

4.2 Ion-Ion interactions

Here provide the pair-wise radial distribution functions of the ion-ion interactions for all ion combinations.

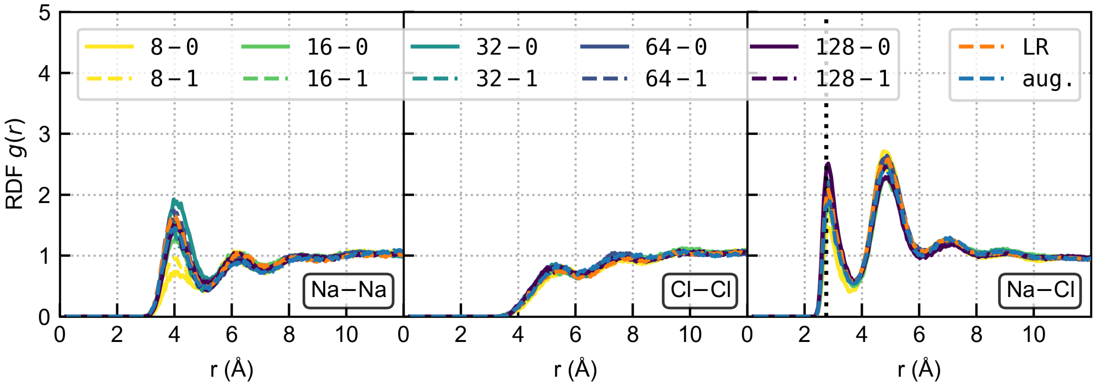

Figure S30: Pairwise radial distribution functions for all three possible ion combinations for all trained MLPs. In addition to the short-range models, results obtained with a long-range corrected model (LR) and from simulations in an enlarged simulation cell (aug.) are shown for comparison. The Na–Cl RDF was employed to obtain the PMF. The experimental reference is taken from Ref. S23.

4.3 Self-diffusion coefficients

The self-diffusion coefficient of all water molecules was computed based on the MSD of the oxygen atoms:

$$D_{\text{self}}^{\text{MD}} = \lim_{t\to\infty} \frac{1}{6t} \left\langle |\boldsymbol{r}(0) - \boldsymbol{r}(t)|^2 \right\rangle = \lim_{t\to\infty} \frac{1}{6t} \left\langle \text{MSD}(t) \right\rangle . \tag{8}$$

with t being the simulation time and $\boldsymbol{r}$ the position of the oxygen atoms.

In practice, $D_{\text{self}}^{\text{MD}}$ was obtained from a linear fit of MSD(t) in the long-time diffusive regime. Unless stated otherwise, the fit window was chosen as $100 \leq t \leq 900$ ps for the 1 ns trajectories, excluding the initial ballistic regime and the final portion of the trajectory, which may be affected by instabilities.

To estimate the statistical uncertainty, we employed block averaging. For each trajectory, the MSD in the fit window ($100 \leq t \leq 900$ ps) was partitioned into blocks of length 100 ps. For each block, a separate linear fit of MSD(t) was performed and converted to a block diffusion coefficient via $D = m/6$, where m corresponds to the slope of the respective segment fit. The reported $D_{\text{self}}^{\text{MD}}$ is the mean over all block estimates pooled across the five independent trajectories for each model.

The statistical uncertainty was quantified using the standard error of the mean of the block diffusion coefficients,

$$\sigma_D = \sqrt{\frac{1}{N_b(N_b-1)} \sum_{b=1}^{N_b} \left(D_b - \overline{D}\right)^2}, \tag{9}$$

where N_b denotes the resulting number of blocks. Trajectories that terminated early contributed only blocks fully contained within the fit window; incomplete blocks were discarded. The resulting averaged self-diffusion coefficients and their resulting uncertainties are shown in the Table below:

Table S3: Block-averaged self-diffusion coefficients of water obtained from molecular dynamics simulations with different MACE model architectures. Reported uncertainties correspond to the combined uncertainty from inter-trajectory variation and intra-trajectory block averaging. N_b denotes the total number of blocks pooled across all trajectories contributing to the estimate.

Model	$D_{\text{self}}^{\text{MD}}$ [10^{-5} cm^2/s]	Uncertainty [10^{-5} cm^2/s]	N_b
8-0	2.89	0.42	28
8-1	2.85	0.12	40
16-0	2.71	0.23	33
16-1	2.83	0.23	40
32-0	2.67	0.16	36
32-1	2.79	0.15	40
64-0	2.75	0.11	40
64-1	2.98	0.22	40
128-0	2.70	0.20	40
128-1	2.67	0.13	40

4.4 Further results of extrapolation study

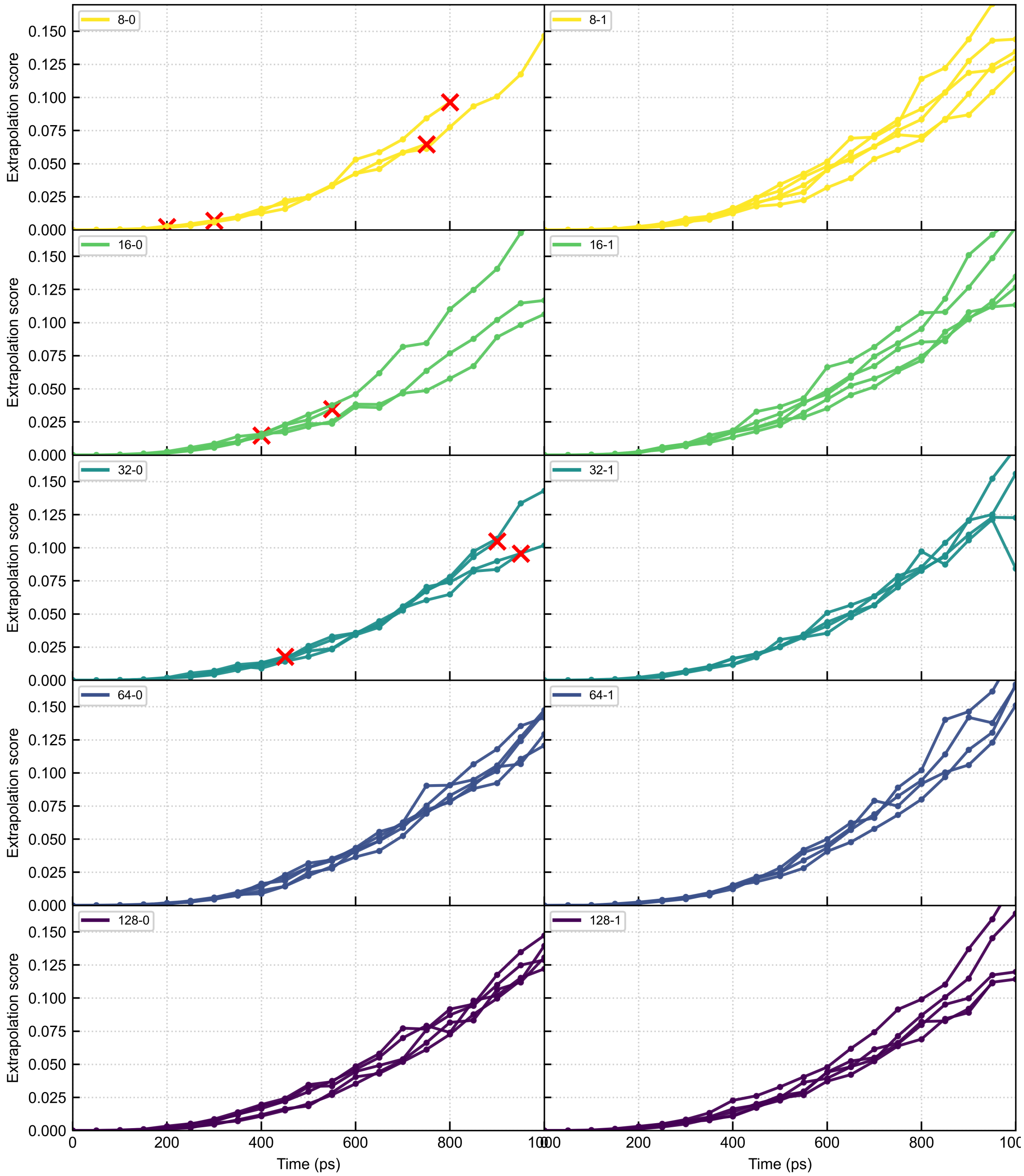

Figure S31: Extrapolation score for all trajectories using the differing MACE architectures. The extrapolation score is computed according to Eq. (2) in the main article. Terminations of the simulation due to unphysical movements are indicated by the red markers.

References

[S1] L. Martínez, R. Andrade, E. G. Birgin, J. M. Martínez, *J. Comput. Chem.* **2009**, *30*, 2157.

[S2] D. Van Der Spoel, E. Lindahl, B. Hess, G. Groenhof, A. E. Mark, H. J. Berendsen, *J. Comput. Chem.* **2005**, *26*, 1701.

[S3] W. L. Jorgensen, J. Tirado-Rives, *J. Am. Chem. Soc.* **1988**, *110*, 1657.

[S4] G. Bussi, D. Donadio, M. Parrinello, *J. Chem. Phys.* **2007**, *126*.

[S5] J. P. Perdew, M. Ernzerhof, K. Burke, *J. Chem. Phys.* **1996**, *105*, 9982.

[S6] C. Adamo, V. Barone, *J. Chem. Phys.* **1999**, *110*, 6158.

[S7] S. Grimme, J. Antony, S. Ehrlich, H. Krieg, *J. Chem. Phys.* **2010**, *132*, 154104.

[S8] J. VandeVondele, J. Hutter, *J. Chem. Phys.* **2007**, *127*.

[S9] S. Goedecker, M. Teter, J. Hutter, *Phys. Rev. B* **1996**, *54*, 1703.

[S10] C. Hartwigsen, S. Gœdecker, J. Hutter, *Phys. Rev. B* **1998**, *58*, 3641.

[S11] M. Krack, *Theor. Chem. Acc.* **2005**, *114*, 145.

[S12] E. Engel, A. Höck, R. Schmid, R. Dreizler, N. Chetty, *Phys. Rev. B* **2001**, *64*, 125111.

[S13] G. Lippert, J. Hutter, M. Parrinello, *Molecular Physics* **1997**, *92*, 477.

[S14] G. Lippert, J. Hutter, M. Parrinello, *Theor. Chem. Acc.* **1999**, *103*, 124.

[S15] M. Iannuzzi, T. Chassaing, T. Wallman, J. Hutter, *Chimia* **2005**, *59*, 499.

[S16] M. Guidon, J. Hutter, J. VandeVondele, *J. Chem. Theory Comput.* **2010**, *6*, 2348.

[S17] N. O'Neill, B. X. Shi, K. Fong, A. Michaelides, C. Schran, *J. Phys. Chem. Lett.* **2024**, *15*, 6081.

[S18] T. D. Kühne, M. Iannuzzi, M. Del Ben, V. V. Rybkin, P. Seewald, F. Stein, T. Laino, R. Z. Khaliullin, O. Schütt, F. Schiffmann, et al., *J. Chem. Phys.* **2020**, *152*, 194103.

[S19] M. Iannuzzi, J. Hutter, *Physical Chemistry Chem. Phys.* **2007**, *9*, 1599.

[S20] I. Batatia, D. P. Kovacs, G. Simm, C. Ortner, G. Csányi, *Advances in Neural Information Processing Systems* **2022**, *35*, 11423.

[S21] J. D. Morrow, J. L. Gardner, V. L. Deringer, *J. Chem. Phys.* **2023**, *158*, 121501.

[S22] D. P. Kovács, I. Batatia, E. S. Arany, G. Csányi, *J. Chem. Phys.* **2023**, *159*, 044118.

[S23] R. Mancinelli, A. Botti, F. Bruni, M. Ricci, A. Soper, *J. Phys. Chem. B* **2007**, *111*,

13570.
